# The fungal α-aminoadipate pathway for lysine biosynthesis requires two enzymes of the aconitase family for the isomerization of homocitrate to homoisocitrate

**DOI:** 10.1111/mmi.12076

**Published:** 2012-11-06

**Authors:** Felicitas Fazius, Ekaterina Shelest, Peter Gebhardt, Matthias Brock

**Affiliations:** 1 Microbial Biochemistry and Physiology, Leibniz Institute for Natural Product Research and Infection Biology, Hans-Knoell-InstituteBeutenbergstr. 11a, 07745, Jena, Germany; 2 Systems Biology/Bioinformatics, Leibniz Institute for Natural Product Research and Infection Biology, Hans-Knoell-InstituteBeutenbergstr. 11a, 07745, Jena, Germany; 3 Cell and Molecular Biology, Leibniz Institute for Natural Product Research and Infection Biology, Hans-Knoell-InstituteBeutenbergstr. 11a, 07745, Jena, Germany

## Abstract

Fungi produce α-aminoadipate, a precursor for penicillin and lysine via the α-aminoadipate pathway. Despite the biotechnological importance of this pathway, the essential isomerization of homocitrate via homoaconitate to homoisocitrate has hardly been studied. Therefore, we analysed the role of homoaconitases and aconitases in this isomerization. Although we confirmed an essential contribution of homoaconitases from *Saccharomyces cerevisiae* and *Aspergillus fumigatus*, these enzymes only catalysed the interconversion between homoaconitate and homoisocitrate. In contrast, aconitases from fungi and the thermophilic bacterium *Thermus thermophilus* converted homocitrate to homoaconitate. Additionally, a single aconitase appears essential for energy metabolism, glutamate and lysine biosynthesis in respirating filamentous fungi, but not in the fermenting yeast *S. cerevisiae* that possesses two contributing aconitases. While yeast Aco1p is essential for the citric acid cycle and, thus, for glutamate synthesis, Aco2p specifically and exclusively contributes to lysine biosynthesis. In contrast, Aco2p homologues present in filamentous fungi were transcribed, but enzymatically inactive, revealed no altered phenotype when deleted and did not complement yeast aconitase mutants. From these results we conclude that the essential requirement of filamentous fungi for respiration versus the preference of yeasts for fermentation may have directed the evolution of aconitases contributing to energy metabolism and lysine biosynthesis.

## Introduction

Fungi synthesize the amino acid lysine *de novo* via the so-called α-aminoadipate pathway (Umbarger, 1978; Bhattacharjee, 1985; Zabriskie and Jackson, 2000). As lysine is an essential amino acid for humans and needs to be obtained from the diet, this pathway has been assumed as a perfect target for the development of new antifungal drugs (Garrad and Bhattacharjee, 1992; Tang *et al*., 1994; Liebmann *et al*., 2004; Xu *et al*., 2006). However, virulence studies showed that the opportunistic human pathogenic fungus *Aspergillus fumigatus* that causes life-threatening invasive aspergillosis only relies on a functional α-aminoadipate pathway for establishment of pulmonal infections (Liebmann *et al*., 2004; Schöbel *et al*., 2010). Studies on disseminated infections from *A. fumigatus* and the dimorphic pathogenic yeast *Candida albicans* revealed that the bloodstream supplies sufficient amounts of lysine to support full virulence of lysine auxotrophic mutants (Kur *et al*., 2010; Schöbel *et al*., 2010; Fleck *et al*., 2011). Thus, it is questioned whether lysine biosynthesis should be further considered as an antifungal drug target.

**Fig. 1:**
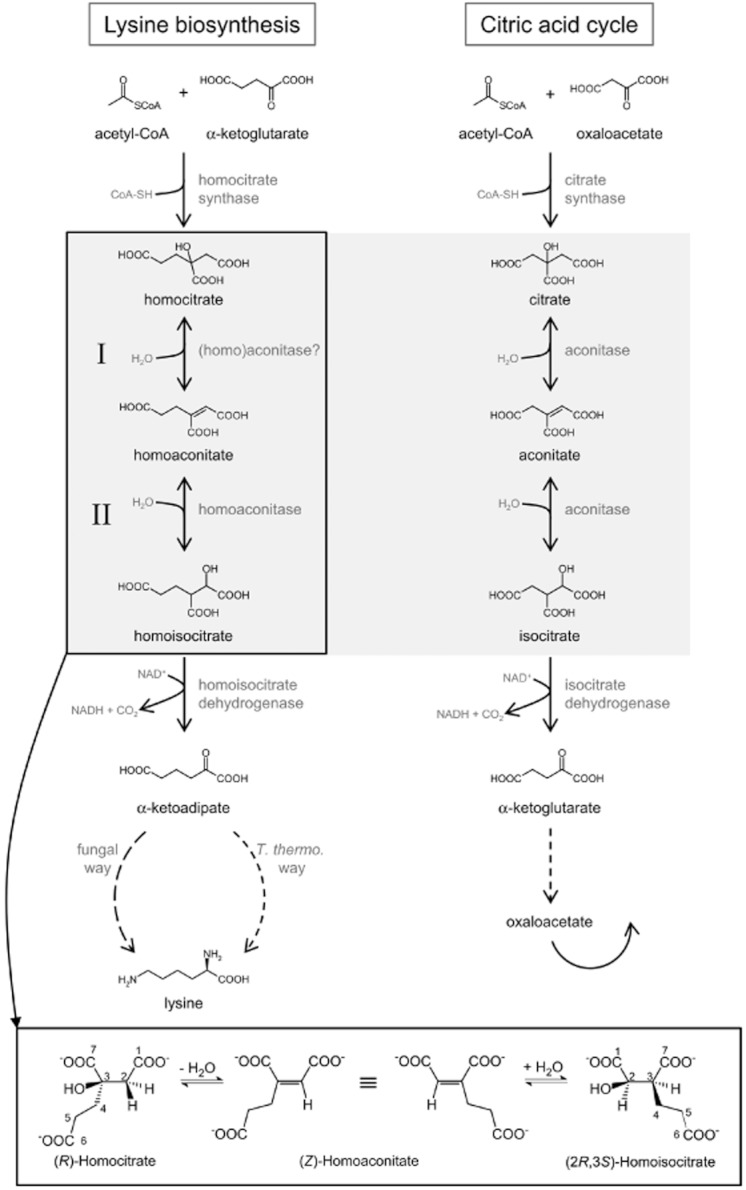
Comparison of the early steps of the α-aminoadipate pathway for lysine biosynthesis with the citric acid cycle. Both pathways require an isomerization of a malate derivative (homocitrate, citrate). In the citric acid cycle, a single enzyme performs the reversible isomerization. The isomerization in the α-aminoadipate pathway is less well studied. Although homoaconitase performs the second reaction (II), its contribution in the homocitrate to homoaconitate dehydration (I) is unclear and could also be performed by an aconitase. The isomerization involving a flip of the substrate in the active site is highlighted in the separate box. Here, (*R*)-homocitrate is dehydrated to form (*Z*)-homoaconitate. After a 180° rotation of homoaconitate (≡) water is added yielding (2*R*, 3*S*)-homoisocitrate.

However, due to the importance of lysine as a food and feed additive this amino acid is produced at industrial scale. Despite the suitability of fungi as lysine producers, lysine production mainly relies on the bacterium *Corynebacterium glutamicum* that synthesizes lysine via the diaminopimelate pathway (Izumi *et al*., 1978; Pfefferle *et al*., 2003). Nevertheless, filamentous fungi not only use the α-aminoadipate pathway for the *de novo* synthesis of lysine, but also require α-aminoadipate for penicillin production (Brakhage, 1998). Here, the internal α-aminoadipate concentration directly correlates with the rate of penicillin production (Jaklitsch *et al*., 1986; Casqueiro *et al*., 1999). Interestingly, despite the importance of the α-aminoadipate pathway for penicillin production, not all enzymatic reactions leading to the formation of α-aminoadipate have been studied in detail, but, at least, all intermediates leading to the formation of α-aminoadipate are known. Synthesis of α-aminoadipate proceeds via the initial condensation of acetyl-CoA and 2-oxoglutarate with the formation of homocitrate (Fig. 1). Homocitrate undergoes dehydration to homoaconitate, which is subsequently rehydrated to homoisocitrate. An oxidative decarboxylation of homoisocitrate forms α-ketoadipate, which is converted to α-aminoadipate by an amino transfer from glutamate. The further steps in lysine biosynthesis involve the formation of α-aminoadipate semialdehyde, saccharopine and, finally, lysine. The latter three reactions are reversible and also used in humans for the degradation of lysine (Fellows and Lewis, 1973).

Interestingly, especially the isomerization of homocitrate via homoaconitate to homoisocitrate has hardly been studied at the enzymatic level. This reaction closely resembles the isomerization of citrate via aconitate to isocitrate in the citric acid cycle (Fig. 1). In the citric acid cycle the isomerization is performed by a single enzyme called aconitase (Lauble *et al*., 1992; 1994; Lauble and Stout, 1995). Detailed analyses on the reaction mechanism of aconitases have shown that the intermediate aconitate can bind in two different modes to the active site. One mode is called the citrate mode, in which water is added to aconitate and forms citrate, whereas the second mode is called isocitrate mode, in which water is added to aconitate and yields isocitrate. To ensure the formation of isocitrate in the latter reaction binding of aconitate in the isocitrate mode is characterized by a 180° ‘flip’ of aconitate within the active site. This ‘flip’ mechanism ensures that during the subsequent reactions of the citric acid cycle, water is first *trans*-eliminated from citrate and subsequently added in *trans* to the rotated aconitate yielding isocitrate (Lauble and Stout, 1995).

In the fungal α-aminoadipate pathway it has been shown that an enzyme called ‘homoaconitase’ essentially contributes to the isomerization of homocitrate to homoisocitrate, as a deletion of the homoaconitase coding gene *lysF* leads to lysine auxotrophy in *Aspergillus* species (Weidner *et al*., 1997; Liebmann *et al*., 2004). However, whether this homoaconitase performs both reactions that are required for the isomerization has not been convincingly shown. Investigations on homoaconitase mutants from the filamentous fungi *Aspergillus nidulans* and *Penicillium chrysogenum* revealed an accumulation of homocitrate, but not homoaconitate (Weidner *et al*., 1997; Teves *et al*., 2009). Thus, it was assumed that homoaconitases from filamentous fungi catalyse both reactions. This assumption was further supported by investigations on a homoaconitase purified and characterized from the methanogenic archaeon *Methanocaldococcus jannaschii* that uses parts of the α-aminoadipate pathway for synthesis of coenzyme B. The heterotetrameric enzyme catalysed both, the dehydration of homocitrate to homoaconitate and the hydration of homoaconitate to homoisocitrate (Drevland *et al*., 2008). Thus, it appeared, at least in principle, possible that homoaconitases perform both reactions.

On the contrary, enzymatic activity determinations and metabolite analyses performed on the yeast *Saccharomyces cerevisiae* pointed to an isomerization involving two independent enzymes. First studies on enriched preparations of homoaconitase from yeast showed that the main product from the hydration of homoaconitate is homoisocitrate, but not homocitrate (Strassman and Ceci, 1966). Nevertheless, some minor amounts of homocitrate were detected and, thus, a hydration of homoaconitate in both directions remained conceivable. However, a lysine auxotrophic mutant unable to convert homoisocitrate into homoaconitate revealed an accumulation of homocitrate and homoaconitate (Maragoudakis, 1967). This clearly pointed to the existence of two independent enzymes involved in the isomerization of homocitrate to homoisocitrate. However, no lysine auxotrophic mutant with a defect in the homocitrate to homoaconitate conversion had been identified.

The separation of this isomerization on two independent enzymes was also supported by studies on the thermophilic bacterium *Thermus thermophilus*. Although bacteria generally utilize the diaminopimelate pathway for lysine biosynthesis, some thermophilic bacteria and archaea possess a modified α-aminoadipate pathway that shares all reactions of α-aminoadipate formation with those of the fungal α-aminoadipate pathway (Kosuge and Hoshino, 1998; 1999). Investigations on purified recombinant homoaconitase from *T. thermophilus* revealed that this enzyme only catalysed the reversible reaction between homoaconitate and homoisocitrate (Jia *et al*., 2006). Interestingly, addition of citric acid cycle aconitase from pig heart completed the isomerization from homocitrate to homoisocitrate (Jia *et al*., 2006). However, in these studies no aconitase had been identified from *T. thermophilus* and, as mammals do not posses a functional α-aminoadipate pathway, it remained speculative whether the combination of aconitase and homoaconitase completes the isomerization under *in vivo* conditions.

Aim of our investigation was to clarify the ambiguous results on the impact of homoaconitase in the isomerization from homocitrate to homoisocitrate in the α-aminoadipate pathway. To address this question, we purified and characterized various aconitases and homoaconitases from filamentous fungi and the yeast *S. cerevisiae*. Additionally, we identified an aconitase from *T. thermophilus* that was tested for its possible contribution to homoisocitrate formation. The impact in lysine biosynthesis of two functional aconitases present in the genome of *S. cerevisiae* was additionally studied by the phenotypic characterizations of mutant strains that were complemented with aconitases from other fungal sources. Results provide evidence that at least one additional aconitase is required for the isomerization of homocitrate, but differences exist between yeast and filamentous fungi. In conclusion, these data are important for the understanding of metabolic physiology and for the optimization of lysine and penicillin production in fungi.

## Results

### Identification, cloning and expression of aconitases from *S. cerevisiae*, *A. nidulans*, *A. fumigatus* and *T. thermophilus*

Aconitases contain an iron–sulphur cluster that is required for the de- and rehydration reaction in the isomerization of citrate to isocitrate in the citric acid cycle (Lauble *et al*., 1992; Beinert *et al*., 1996; Lloyd *et al*., 1999). Oxidation of the [4Fe–4S]-cluster in the presence of air leads to loss of a labile iron and subsequent loss of the complete cluster inactivating the enzyme (Flint *et al*., 1993; Beinert *et al*., 1996). To overcome these problems, we selected a strategy by producing and purifying recombinant apoenzymes in *Escherichia coli* with subsequent reintroduction of the iron–sulphur cluster.

*Saccharomyces cerevisiae* contains two actively transcribed aconitases called Aco1p and Aco2p (Gangloff *et al*., 1990; Przybyla-Zawislak *et al*., 1999). Aco1p is the major aconitase from the citric acid cycle and an *aco1* deletion mutant is viable on glucose, but requires the presence of glutamate (Gangloff *et al*., 1990). In contrast, the role of Aco2p remains unknown (Przybyla-Zawislak *et al*., 1999). Both aconitase genes contain no intron sequences and were directly amplified from genomic DNA without the region coding for the mitochondrial import sequence and cloned into a pET expression vector with N-terminal His-tag (Hortschansky *et al*., 2007).

**Fig. 2:**
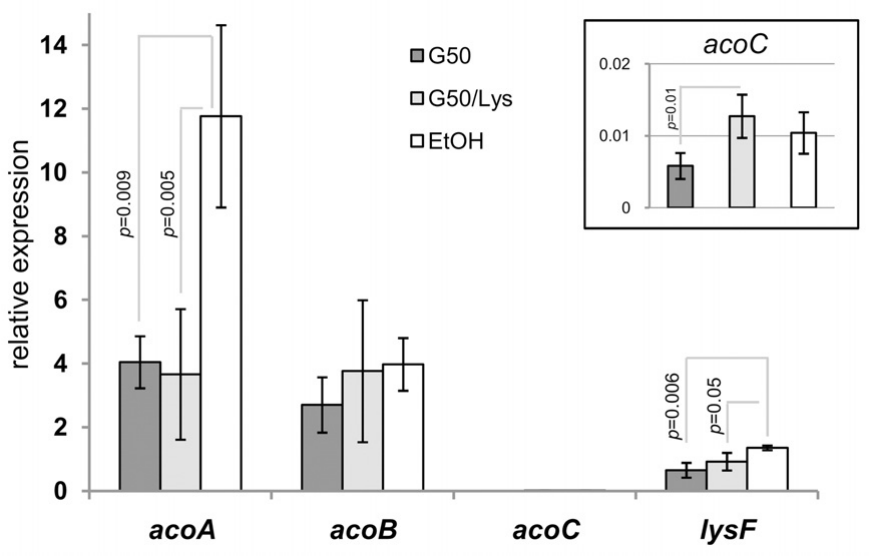
Expression analyses of *A. fumigatus* aconitases and homoaconitase by qRT-PCR. Expression of *A. fumigatus* aconitases *acoA*, *acoB*, *acoC* and homoaconitase *lysF* normalized against tubulin gene expression. Cells were grown on glucose minimal medium (G50), glucose medium with lysine (G50/Lys) and ethanol minimal medium (EtOH). Data show mean values of three independent experiments performed in technical triplicates. Statistical significance was calculated by the paired *t*-test. *acoA* expression is significantly induced during growth on ethanol, which is in line with its contribution to the glyoxylate cycle. Addition of lysine to glucose medium does not alter expression levels. *acoB* is constitutively expressed under all three conditions. Expression of *acoC* remained near the detection limit and is displayed in the inlet of the figure. Similar to *acoA*, expression of *lysF* is not repressed by lysine but slightly increases during growth on ethanol.

Similar to *S. cerevisiae*, at least two genes coding for putative aconitases are present in the genome of *A. fumigatus*. Aconitases from Aspergilli have not been characterized in detail and it remained speculative which of the enzymes contributes to aconitase activity. Thus, we partially purified the *A. fumigatus* aconitase from glucose-grown cells using aconitate hydratase activity as a readout system. After hydrophobic interaction chromatography, anionic exchange chromatography and a final chromatography on Reactive Red agarose, we ended up with an active fraction that showed four major protein bands in SDS-PAGE analysis (data not shown). These bands were subjected to tryptic digestion and subsequent MALDI-TOF analysis. By this method, only AcoA (protein accession XP_751171) and no other aconitase-like protein was identified implying that this protein is the major aconitase in *A. fumigatus*. AcoA consists of 787 amino acids with a 33-amino-acid mitochondrial import sequence. cDNA was generated from total RNA and the coding sequence without mitochondrial signal peptide was used for recombinant protein production in *E. coli*. Similarly, a highly identical AcoA protein from *A. nidulans* (protein accession XP_663129; 90% sequence identity to *A. fumigatus* AcoA) that has been assumed to act as the main citric acid cycle aconitase (Oberegger *et al*., 2002) was cloned for recombinant enzyme production. qRT-PCR analysis on *A. fumigatus acoA* (Fig. 2) revealed constitutive expression on glucose medium with or without lysine and increased expression on ethanol, which is in agreement with an additional requirement of aconitase activity to serve for the glyoxylate cycle.

Although only AcoA was identified from our purification procedure, qRT-PCR analyses revealed that the second aconitase-like enzyme, AcoB, from *A. fumigatus* (protein accession XP_750430) also showed constitutive expression levels on glucose minimal medium with or without lysine and during growth on ethanol (Fig. 2). AcoB proteins from *A. fumigatus* and *A. nidulans* (protein accession XP_661498) are 53% identical to their respective AcoA protein and were also selected for recombinant protein production. As the *acoB* genes from *A. fumigatus* and *A. nidulans* only contained a single intron within the mitochondrial import sequence, both genes were amplified without mitochondrial leader sequence from genomic DNA and cloned into the pET expression vector.

A third aconitase-like enzyme called AcoC (protein accession XP_747512) was identified from the genome of *A. fumigatus*. However, AcoC only showed 15% sequence identity to AcoA and analysis of gene expression revealed that expression levels hardly exceeded threshold values (Fig. 2). As expression of the homoaconitase gene *lysF* was significantly higher than that of *acoC* (Fig. 2), the latter was not studied further. Interestingly, *lysF* expression was not suppressed in the presence of lysine, but showed a slight but significant increase compared to glucose medium without lysine, which is in agreement with expression studies on the homocitrate synthase *hcsA* (Schöbel *et al*., 2010). This confirms that, in contrast to *S. cerevisiae*, lysine does not act as a strong feedback inhibitor of the α-aminoadipate pathway in *A. fumigatus*. Unexpectedly, *lysF* expression also increased when cells were grown on ethanol as sole nutrient source. However, this phenomenon was not further followed.

Finally, we looked for an aconitase from the thermophilic bacterium *T. thermophilus*. A protein annotated as aconitate hydratase (protein accession YP_143992) was identified and the gene was amplified from genomic DNA and cloned into the pET expression vector for recombinant production in *E. coli*.

In summary, seven aconitase genes were cloned in pET expression vectors to produce proteins with N-terminal His-tag. All proteins were produced in soluble form as confirmed by SDS-PAGE analysis (see Fig. S1). Purification was performed by chromatography on Ni-chelate columns and, in case of *S. cerevisiae* Aco1p and *Aspergillus* AcoB proteins, by additional use of anionic exchange chromatography. All aconitases, except that of the highly thermostable aconitase from *T. thermophilus*, were enzymatically inactive after purification, which was in agreement with a partial or complete loss of the [4Fe–4S]-cluster.

### Recombinant production of homoaconitase from *S. cerevisiae* and *A. fumigatus*

Similar to aconitases, homoaconitases seem to contain an iron–sulphur cluster required for enzymatic activity (Irvin and Bhattacharjee, 1998). Although fungal homoaconitases have not been purified and characterized before, sequence identity to the iron–sulphur cluster containing homoaconitase from *T. thermophilus* (Jia *et al*., 2006) and the presence of conserved cysteine residues possibly involved in cluster formation (Lauble and Stout, 1995; Irvin and Bhattacharjee, 1998) strengthened this assumption. Unfortunately, in cell-free extracts of fungi, activities of enzymes involved in lysine biosynthesis are rather low (Strassman and Ceci, 1965; 1966; Ramos *et al*., 1988; Weidner *et al*., 1997), which renders purification from the native source extremely difficult. Thus, as for the purification of aconitases, we selected a strategy of recombinant protein production in the heterologous host *E. coli*.

The Lys4p (protein accession NP_010520) has been described to act as homoaconitase (homoaconitate hydratase) in *S. cerevisiae* (Bhattacharjee *et al*., 1968; Zabriskie and Jackson, 2000) and the gene was amplified from genomic DNA without the sequence coding for the mitochondrial leader peptide and cloned into the pET expression vector. To analyse whether homoaconitases from yeasts and filamentous fungi possess the same substrate specificity, we additionally selected the *lysF* gene from *A. fumigatus* (Liebmann *et al*., 2004) for recombinant protein production (protein accession CAC48042). The *lysF* gene was amplified without mitochondrial leader peptide sequence from cDNA and cloned into the pET expression vector. Both proteins were purified by Ni-chelate chromatography (Fig. S1). Purified enzymes showed no activity with homoaconitate as substrate, implying that the enzymes were purified as apoenzymes without an intact iron–sulphur cluster.

### Reactivation and activity determination of aconitases and homoaconitases

Due to the inactivity of all purified aconitases and homoaconitases, with the exception of the thermostable *T. thermophilus* aconitase, the iron–sulphur cluster was reconstituted under reducing anaerobic conditions similar to a previously described method (Albrecht *et al*., 2010). Reactivated proteins were stored at −80°C and freshly thawed aliquots were used for activity determinations. Measurements from thawed samples were possible for a maximum of 30 min, because repeated measurements indicated a rapid inactivation in the presence of air (Table S1).

In order to determine the general substrate specificity of purified enzymes, activities were determined by monitoring changes in absorbance at 240 nm. This method allows visualizing re- or dehydration reactions of the substrates aconitate, homocitrate, homoaconitate and homoisocitrate. However, this method cannot distinguish whether (homo)aconitate substrates are hydrated into the direction of (homo)citrate or (homo)isocitrate. A summary of the results from activity determinations is shown in Table 1.

**Table 1:** Specific activities of recombinant *S. cerevisiae* and *A. fumigatus* aconitases, homoaconitases and *S. cerevisiae* Lys12p

Enzyme	Citrate	Aconitate	Isocitrate	Homocitrate	Homoaconitate	Homoisocitrate
ScAco1p	4.04	23.7	9.65	n.d.	0.9	n.d.
ScAco2p	0.017	0.005	0.043	n.d.	4.76	n.d.
AfAcoA	3.9	12.3	5.15	n.d.	2.7	n.d.
AnAcoA	3.32	14.2	7.15	n.d.	2.1	n.d.
AfAcoB	0.037	n.d.	0.044	–	n.d.	–
AnAcoB	0.033	n.d.	0.042	–	n.d.	–
TtAcoA^a^	1.48	(1.6) 4.73	2.09	n.d.	(0.05) 0.63	n.d.
Lys4p	0.02	0.022	0.024	n.d.	0.29	0.5
LysF	0.99	0.017	1.88	n.d.	2.7	7.5
ScLys12p	0.028	n.d.	0.023	0.003	n.d.	0.81

Specific activities of iron–sulphur cluster containing enzymes depict mean values from three independent freshly thawed aliquots. Deviations between samples were < 10%; all activities are given in [U mg].

a Values for TtAcoA in brackets = measurement at 22°C, other values determined at 60°C.

n.d., not detectable; –, not tested.

As expected, both homoaconitases were active with homoisocitrate and homoaconitate, indicating that the reversible reaction between these two intermediates is indeed catalysed by homoaconitases. However, when homocitrate was used as a substrate, activity was neither observed with the yeast nor with the *A. fumigatus* enzyme. Thus, homoaconitases seem unable to dehydrate homocitrate. Alternatively, the equilibrium of the reaction is far on the side of homocitrate making the formation of homoaconitate difficult to observe.

Similar to homoaconitases, all reactivated aconitases that were active with aconitate as substrate (*A. fumigatus* AcoA, *A. nidulans* AcoA, *T. thermophilus* AcoA, *S. cerevisiae* Aco1p and Aco2p) hydrated homoaconitate (Table 1), but, unexpectedly, activity was neither observed with homocitrate nor with homoisocitrate.

Interestingly, Aco2p was highly active with homoaconitate and displayed a 950-fold higher specific activity with homoaconitate than with aconitate. In contrast, Aco1p, the major citric acid cycle aconitase (Gangloff *et al*., 1990), was 26-fold more active with aconitate than with homoaconitate. Similar to Aco1p, the aconitases AcoA from *A. fumigatus* and *A. nidulans* and the aconitate hydratase from *T. thermophilus* displayed higher activities with aconitate than with homoaconitate, although differences for the latter enzymes were less strongly pronounced than for *S. cerevisiae* Aco1p (Table 1). Unexpectedly, no activity was observed with the second aconitase (AcoB) from *A. fumigatus* and *A. nidulans*. This observation implied that especially in *S. cerevisiae* Aco2p plays a specific role in the α-aminoadipate pathway. However, at this point, we were unable to exclude that production of correctly folded enzymes in *E. coli* failed.

### Identification and recombinant production of homoisocitrate dehydrogenase from *A. fumigatus* and *S. cerevisiae*

For *in vitro* reconstruction of the homocitrate to homoisocitrate isomerization, it appeared essential to remove homoisocitrate from the reaction equilibrium. Within the α-aminoadipate pathway this is performed by the decarboxylating activity of the homoisocitrate dehydrogenase (Lin *et al*., 2007). Thus, we aimed in the recombinant production of the proposed homoisocitrate dehydrogenase LysB from *A. fumigatus* (Xue *et al*., 2004) and Lys12p from *S. cerevisiae* (Lin *et al*., 2007).

Although a deletion of *lysB* in *A. fumigatus* resulted in strongly reduced growth on media without lysine supplementation and, additionally, *lysB* complemented the lysine auxotrophic phenotype of a *S. cerevisiae lys12* mutant (Fig. S2), all approaches to produce LysB as an active recombinant protein in *E. coli* failed. Despite the use of N- or C-terminal His-tags, cloning without tag or as a MalE fusion, only insoluble or inactive protein fractions were obtained (data not shown). Therefore, we only produced recombinant Lys12p by a procedure previously described (Lin *et al*., 2007). The protein was purified by Ni-chelate chromatography (Fig. S1J) and showed a specific activity of 0.81 U mg^−1^ with homoisocitrate as substrate (Table 1). Activities with isocitrate or homocitrate remained near the detection limit, which confirmed the substrate specificity of this enzyme (Yamamoto *et al*., 2007).

### *In vitro* formation of α-ketoadipate from homocitrate

**Fig. 3:**
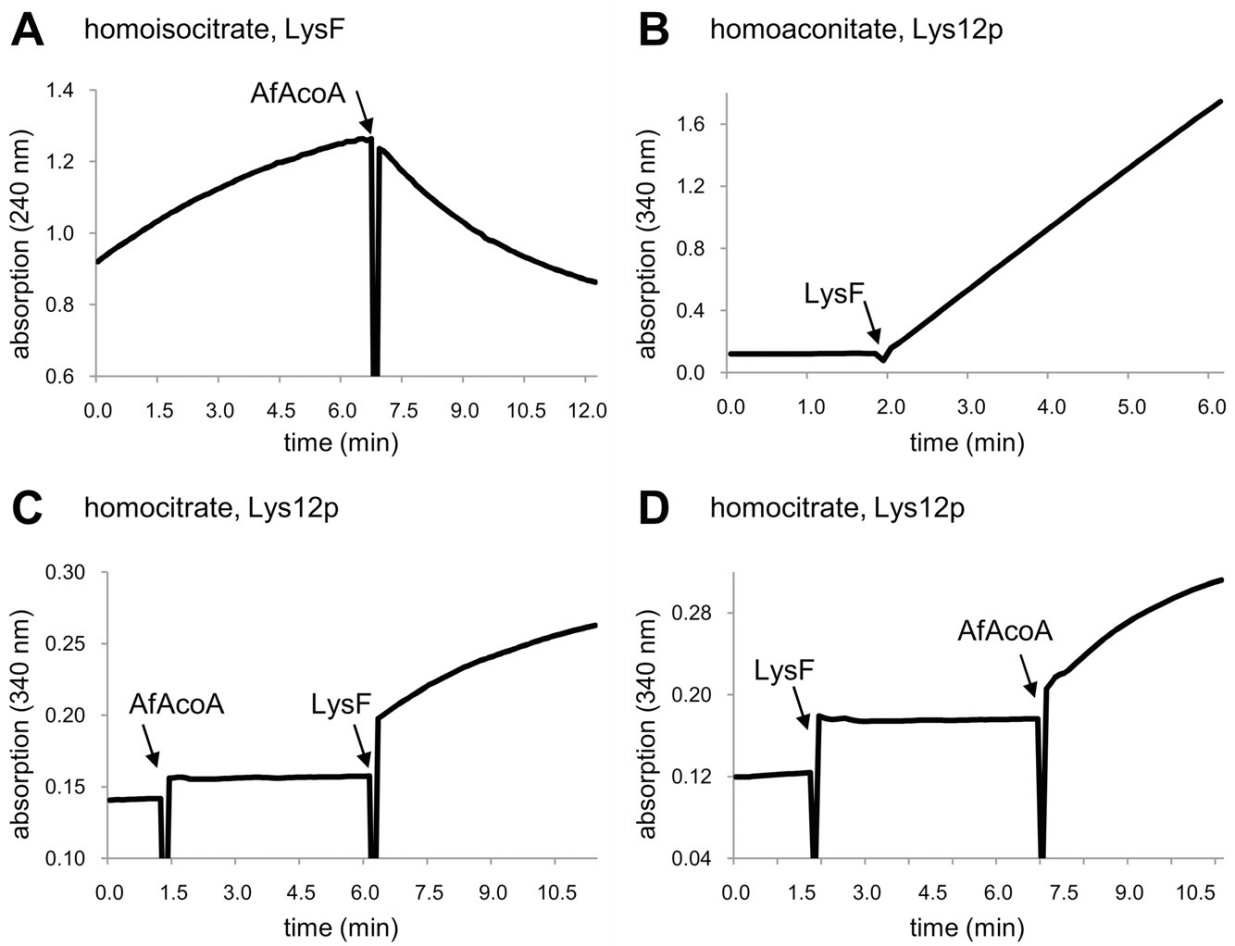
Spectrophotometric determination of substrate conversion by aconitase and homoaconitase. Depicted are example determinations for *A. fumigatus* homoaconitase (LysF) and aconitase (AfAcoA) in combination with the *S. cerevisiae* homoisocitrate dehydrogenase Lys12p. A. Homoisocitrate in the combination with LysF leads to an increase of absorption at 240 nm pointing to the formation of homoaconitate. Addition of AfAcoA (arrow) leads to a decrease in absorption, which indicates the hydration of homoaconitate to homocitrate. B. Homoaconitate was first incubated with Lys12p in a NAD-dependent assay. No change in absorbance at 340 nm confirms that Lys12p is inactive with this substrate. Addition of homoaconitase LysF (arrow) leads to a strong increase in absorbance at 340 nm due to the formation of homoisocitrate, which is subsequently decarboxylated by Lys12p. C. Homocitrate was first incubated with Lys12p in a NAD-dependent assay. No change in absorbance confirms that Lys12p is inactive with homocitrate. Addition of aconitase (first arrow) does not increase absorption. Subsequent addition of LysF (second arrow) leads to an ongoing increase in absorption due to the formation of homoisocitrate that becomes decarboxylated by Lys12p. D. Preincubation of homocitrate with Lys12p and subsequent addition of LysF (first arrow). No change in absorbance indicates the inability of LysF to isomerize homocitrate into homoisocitrate. Subsequent addition of AfAcoA (second arrow) leads to increase in absorption confirming the requirement of the combined action of aconitase and homoaconitase in the isomerization reaction.

For *in vitro* reconstruction of the homocitrate to homoisocitrate isomerization and to elucidate the substrate specificity of the involved enzymes, we used different combinations of aconitases, homoaconitases and homoisocitrate dehydrogenase in conjunction with different substrates. Examples for the spectrophotometric activity determinations are shown in Fig. 3.

At first, we used a combination of LysF and homoisocitrate with subsequent addition of *A. fumigatus* AcoA (Fig. 3A). Here, LysF dehydrated homoisocitrate and formed homoaconitate as indicated by an increase of absorption at 240 nm. Subsequent addition of aconitase AcoA decreased absorption as expected for the formation of homocitrate. However, when homocitrate was incubated first with AcoA and subsequently with LysF, no change in absorbance was observed (not shown). This implies that indeed the equilibrium of the reaction is far on the side of homocitrate. When homoaconitate, but not homocitrate, was used as substrate and LysF was used in combination with the homoisocitrate dehydrogenase Lys12p, a reduction of NAD was detected at 340 nm, which indicates the formation of α-ketoadipate and confirms the formation of homoisocitrate from homoaconitate by LysF (Fig. 3B). In contrast, when the aconitase AcoA was used in combination with Lys12p, neither homocitrate nor homoaconitate led to a reduction of NAD (not shown). This confirms that AcoA is unable to produce homoisocitrate from homoaconitate. However, when homocitrate served as substrate, the combination of AcoA, LysF and Lys12p resulted in a reduction of NAD (Fig. 3C and D). Similarly, when AcoA from *A. fumigatus* was replaced by *A. nidulans* AcoA, the *T. thermophilus* aconitate hydratase or by the *S. cerevisiae* aconitases Aco1p or Aco2p, homocitrate was converted to homoisocitrate in the presence of *A. fumigatus* LysF or *S. cerevisiae* homoaconitase Lys4p (not shown). No activity was observed when one of the aconitases was replaced with *A. fumigatus* or *A. nidulans* AcoB. Thus, at least under *in vitro* conditions, an active aconitase in combination with a homoaconitase is essential for the isomerization of homocitrate to homoisocitrate. However, the question remained open, whether these *in vitro* observations were also valid under *in vivo* conditions.

### Characterization of *S. cerevisiae* *aco1*, *aco2* and *aco1/aco2* mutants

**Fig. 4:**
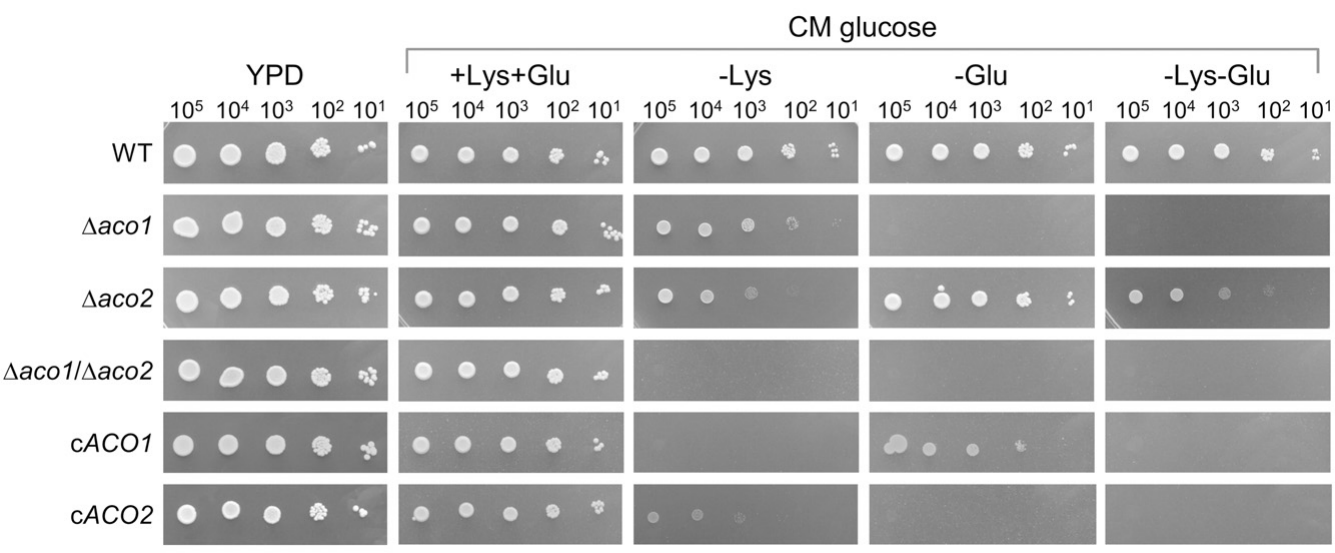
Growth analysis of *S. cerevisiae* aconitase mutants. Spot dilution test on solid media. WT = wild type, Δ*aco1* = *aco1* deletion mutant, Δ*aco2* = *aco2* deletion mutant, Δ*aco1*/Δ*aco2* = mutant deleted in the *aco1* and *aco2* gene, c*ACO1* = Δ*aco1*/Δ*aco2* mutant complemented with the *ACO1* gene, c*ACO2* = Δ*aco1*/Δ*aco2* mutant complemented with the *ACO2* gene. All strains grow with similar efficiency on YPD medium and on glucose medium supplemented with lysine and glutamate (+Lys +Glu). In the absence of lysine (−Lys) growth of Δ*aco1* and Δ*aco2* is retarded, while the Δ*aco1*/Δ*aco2* is strictly lysine-dependent. Without glutamate (−Glu) mutants deleted in the *aco1* gene are unable to grow. Without lysine and glutamate (−Lys −Glu) neither the double mutant nor the Δ*aco1* mutant is able to grow. Reintroduction of the *ACO1* gene in the double mutant (c*ACO1*) restores glutamate auxotrophy, whereas reintroduction of the *ACO2* gene (c*ACO2*) mainly restores lysine auxotrophy.

Yeasts are generally able to ferment glucose. Under such conditions the citric acid cycle is mainly required for the anaplerotic provision of biosynthetic building blocks such as α-ketoglutarate. Therefore, as long as glutamate is provided in the growth medium, *S. cerevisiae aco1* mutants are able to grow on glucose without obvious phenotypes. Thus, the glutamate auxotrophy of *aco1* mutants indicates that Aco1p is the major citric acid cycle aconitase in *S. cerevisiae* (Gangloff *et al*., 1990). In contrast, no relevant phenotype had been described for an *aco2* mutant. As we assumed from our *in vitro* studies that aconitases essentially contribute to lysine biosynthesis, we obtained *aco1* and *aco2* mutants from a mutant library and constructed an *aco1*/*aco2* double mutant strain. All strains were tested for their dependence on glutamate and lysine in the growth medium (Fig. 4).

As expected, all mutants with an *aco1* deletion required the addition of glutamate to the medium, but on complete minimal glucose or YPD medium no growth defects were observed. However, although the *aco1* and the *aco2* mutants grew on glutamate containing minimal medium without lysine, growth rate in comparison to the parental strain was reduced for both mutants. In addition, this phenotype was more strongly pronounced for the *aco2* mutant. The most severe phenotype was observed for the *aco1*/*aco2* double mutant. This strain was completely auxotrophic for lysine and in its absence growth was observed neither on plates nor in liquid medium.

To confirm that lysine and glutamate auxotrophy in the double deletion mutant was the direct result of the deletion of *ACO2* in the *aco1* negative background, both genes were independently reintroduced in the original genomic locus. Reintroduction of *ACO1* (c*ACO1*) restored glutamate auxotrophy, but growth in the absence of lysine remained strongly retarded. In contrast, reintroduction of *ACO2* in the double deletion mutant (c*ACO2*) retained glutamate auxotrophy, but lysine dependence was less pronounced (Fig. 4). These results indicated that Aco1p mainly serves for the citric acid cycle with minor contribution to lysine biosynthesis, whereas Aco2p does not contribute to the citric acid cycle but is specifically involved in the α-aminoadipate pathway. This is also in agreement with the high *in vitro* activity of Aco2p with homoaconitate compared to its low activity with aconitate (Table 1).

### Expression analysis of *S. cerevisiae* *ACO1* and *ACO2*

**Fig. 5:**
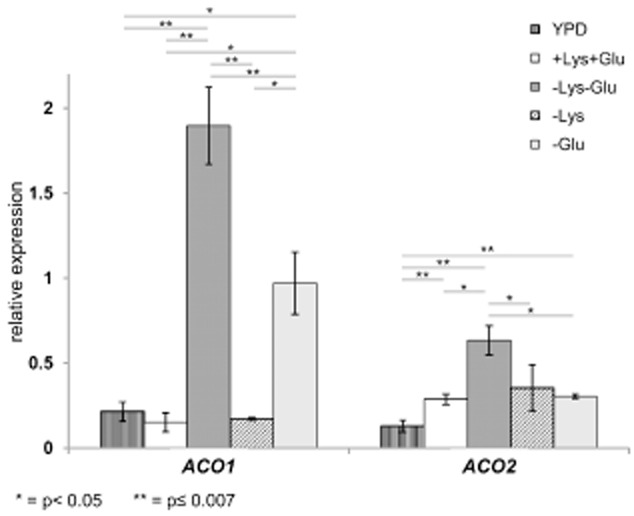
Expression analysis of *S. cerevisiae* *ACO1* and *ACO2* by qRT-PCR. Tubulin expression was used for standardization. Relative expression was compared between the following media: YPD, glucose medium with lysine and glutamate (+Lys +Glu), glucose medium without lysine and glutamate (−Lys −Glu), glucose medium without lysine but with glutamate (−Lys) and glucose medium without glutamate but with lysine (−Glu). Data show mean values of three independent experiments performed in technical triplicates. Statistical significance was calculated by the paired *t*-test. For a detailed explanation, refer to the main text.

As both *S. cerevisiae* aconitases contributed to varying extent to lysine biosynthesis, we investigated the expression pattern of these aconitases in respect to the available amino acids in the growth medium. The wild-type strain CLIB 334 was freshly grown in YPD medium, washed and transferred either back to YPD medium or to complete minimal media with glutamate and lysine or lacking one or both of these amino acids. Transcription levels were determined by real-time PCR using the *S. cerevisiae TUB2* gene (YFL037w) for normalization of expression levels (Fig. 5). Analyses revealed that *ACO1* expression was strongly induced in the absence of glutamate, but not when only lysine was lacking. Nevertheless, when glutamate and lysine were both lacking from the growth medium, *ACO1* expression further increased. This could be due to the increased requirement of α-ketoglutarate not only for glutamate synthesis, but also for the synthesis of homocitrate via homocitrate synthase in fungal lysine biosynthesis (Schöbel *et al*., 2010). In conclusion, this transcriptional profile confirms the function of Aco1p as main aconitase in the citric acid cycle and is in agreement with the glutamate auxotrophy of *aco1* mutants.

In contrast to *ACO1* expression, the expression profile of *ACO2* was not clearly attributable to the medium composition, although *ACO2* expression was lower on YPD medium than on complete minimal media. However, expression levels on minimal media with both amino acids present, only lacking lysine or only lacking glutamate showed no statistically significant differences. However, significant induction was observed on media lacking both amino acids, but expression levels only increased by a factor of about two compared to the same medium with both amino acids present. This analysis shows that *ACO1* and *ACO*2 expression patterns are not directly linked, as *ACO2* expression appears constitutively active (although at a low level), whereas *ACO1* expression is strongly dependent on the availability of glutamate.

### Aconitase requirements of *A. fumigatus*

It can be assumed that *A. fumigatus* cannot grow in an exclusively fermentative manner (Taubitz *et al*., 2007; Barker *et al*., 2012) and requires the citric acid cycle to gain energy from oxidative decarboxylation reactions and the respiratory chain. To test the contribution of *A. fumigatus* AcoA and AcoB for growth, lysine and glutamate production, we aimed in the construction of the respective deletion mutants.

**Fig. 6:**
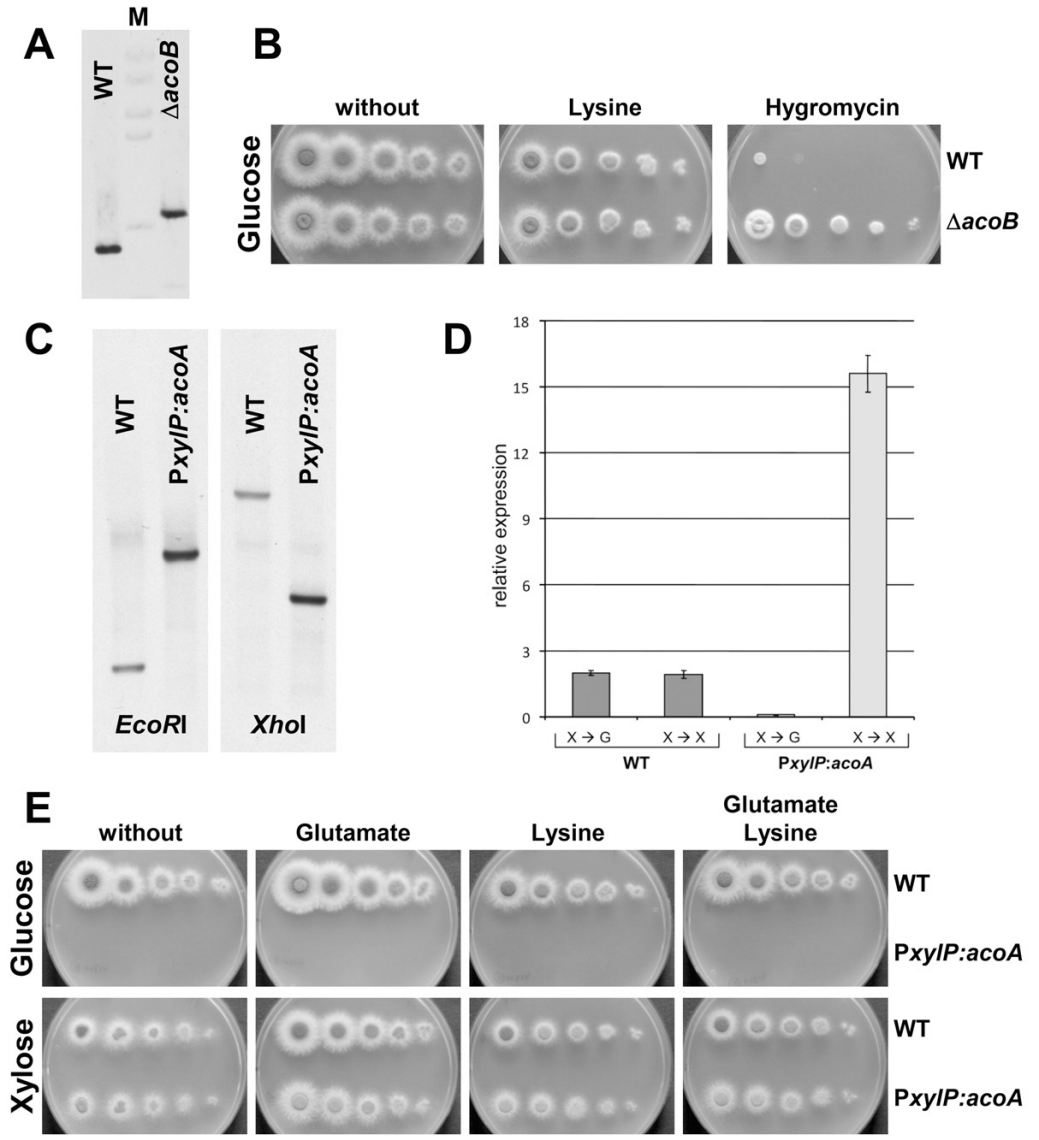
Characterization of an *A. fumigatus* *acoB* deletion mutant (Δ*acoB*) and of a strain carrying an exchange of the *acoA* promoter by the xylose-inducible *xylP* promoter (P*xylP:acoA*). For growth analyses conidia were diluted in the range between 1 × 10^5^ and 10 conidia and spot-inoculated on plates. A. Southern blot analysis for confirmation of *A. fumigatus* *acoB* gene deletion from genomic DNA restricted with SalI. The expected shift of the signal due to introduction of the hygromycin B resistance cassette at the *acoB* locus is visible. B. Growth of wild type (WT) and *acoB* deletion mutant (Δ*acoB*) on glucose medium with and without lysine and in the presence of hygromycin B. The hygromycin B-resistant *acoB* mutant displays no delayed growth in the absence of lysine. C. Southern analysis of a transformant with replacement of the *acoA* promoter by the *xylP* promoter. Genomic DNA was either restricted with EcoRI (expected shift: 2.2 → 4.9 kb) or XhoI (expected shift: 9.1 → 3.6 kb) and hybridized with a probe against the *acoA*-coding region. D. qRT-PCR transcript analyses on *acoA* from *A. fumigatus* wild type and promoter exchange mutant after a 7 h shift from xylose to glucose (X → G) or xylose to xylose (X → X). Data were normalized against tubulin expression and determined in technical triplicates. Error bars denote the standard deviation. The same samples were used for aconitase activity determinations as described in the main text. In the wild-type strain *acoA* transcript levels remain constant regardless of the shift to glucose or xylose. In the P*xylP:acoA* strain *acoA* is strongly transcribed during growth on xylose, but transcripts are hardly detectable after a shift to glucose. E. Growth analyses of wild type and P*xylP:acoA* strain on glucose (upper plates) or xylose (bottom plates) with or without amino acids. P*xylP:acoA* is unable to germinate on glucose regardless of the presence of amino acids, but grows comparable to the wild type on xylose medium.

To study the impact of *acoB* we replaced the *acoB* gene by the hygromycin B resistance cassette as confirmed by Southern blot analysis (Fig. 6A). When the mutant strain was analysed for growth phenotypes on agar plates containing glucose with and without lysine, no altered phenotypes were observed in comparison to the parental strain (Fig. 6B). Furthermore, when cultivated under hypoxic conditions or in the presence of varying amounts of iron, no different phenotype was observed (data not shown). Thus, these analyses support our *in vitro* data on purified recombinant AcoB proteins that revealed no activity with any of the substrates tested and, unlike Aco2p from *S. cerevisiae*, appear of minor importance for normal growth or lysine biosynthesis.

In contrast to the *acoB* deletion, we were unable to delete the *acoA* gene. Several attempts to replace the *acoA*-coding region by either the hygromycin B or pyrithiamine resistance cassette failed and only yielded transformants with ectopic integrations (data not shown). However, a strategy was selected in which we replaced the endogenous *acoA* promoter by the *xylP* promoter from *P. chrysogenum*. This promoter has been shown to be silent when cells are grown on glucose, but is highly induced in the presence of xylose (Zadra *et al*., 2000). As confirmed by PCR (not shown) and Southern blot analysis, a strain was identified which carried the desired promoter replacement (Fig. 6C). When this strain was cultivated on xylose, normal colony formation was observed and, compared to the parental strain, glutamate or lysine supplementation had no beneficial or detrimental effects (Fig. 6E). In contrast, when the same strains were cultivated on glucose and regardless of the supplementation of glutamate or lysine, only the parental wild type, but not the promoter exchange mutant was able to grow. To confirm that this phenotype was due to reduced *acoA* expression, a carbon source shift experiment was performed. Both strains were pre-cultivated on liquid xylose medium, harvested, washed and either transferred back to xylose or shifted to glucose medium. After 7 h of incubation, mycelia were harvested and used for activity determination and *acoA* expression analyses. For cells that were shifted back to xylose the P*xylP:acoA* strain showed a specific aconitase activity of 3055 ± 52 mU mg^−1^, whereas the wild type displayed only 252 ± 22 mU mg^−1^. When shifted to glucose, activity of the wild-type strain remained constant (265 ± 10 mU mg^−1^). In contrast, aconitase activity of the P*xylP:acoA* was reduced on glucose by a factor of two (1457 ± 40 mU mg^−1^). However, this activity was still much higher (approximately factor 6) than that of the wild type and implicated that *acoA* expression either remained at high levels or that the intracellular stability of AcoA was responsible for this high level of activity. Therefore, qRT-PCR was performed on the same samples (Fig. 6D). While *acoA* expression in the wild type maintained at a constant level regardless of the growth on glucose or xylose, *acoA* expression in the promoter exchange mutant was eight times higher than in the wild type on xylose, but 21 times lower than in the wild type when shifted to glucose. Therefore, the inability of the promoter exchange mutant to germinate on glucose medium is due to a lack of *acoA* expression. Furthermore, as lysine and glutamate supplementation did not restore growth of the mutant on glucose (Fig. 6E), it can be concluded that AcoA is essentially required for energy metabolism.

### Complementation of the *S. cerevisiae* *aco1/aco2* double mutant with *Aspergillus* aconitases

**Fig. 7:**
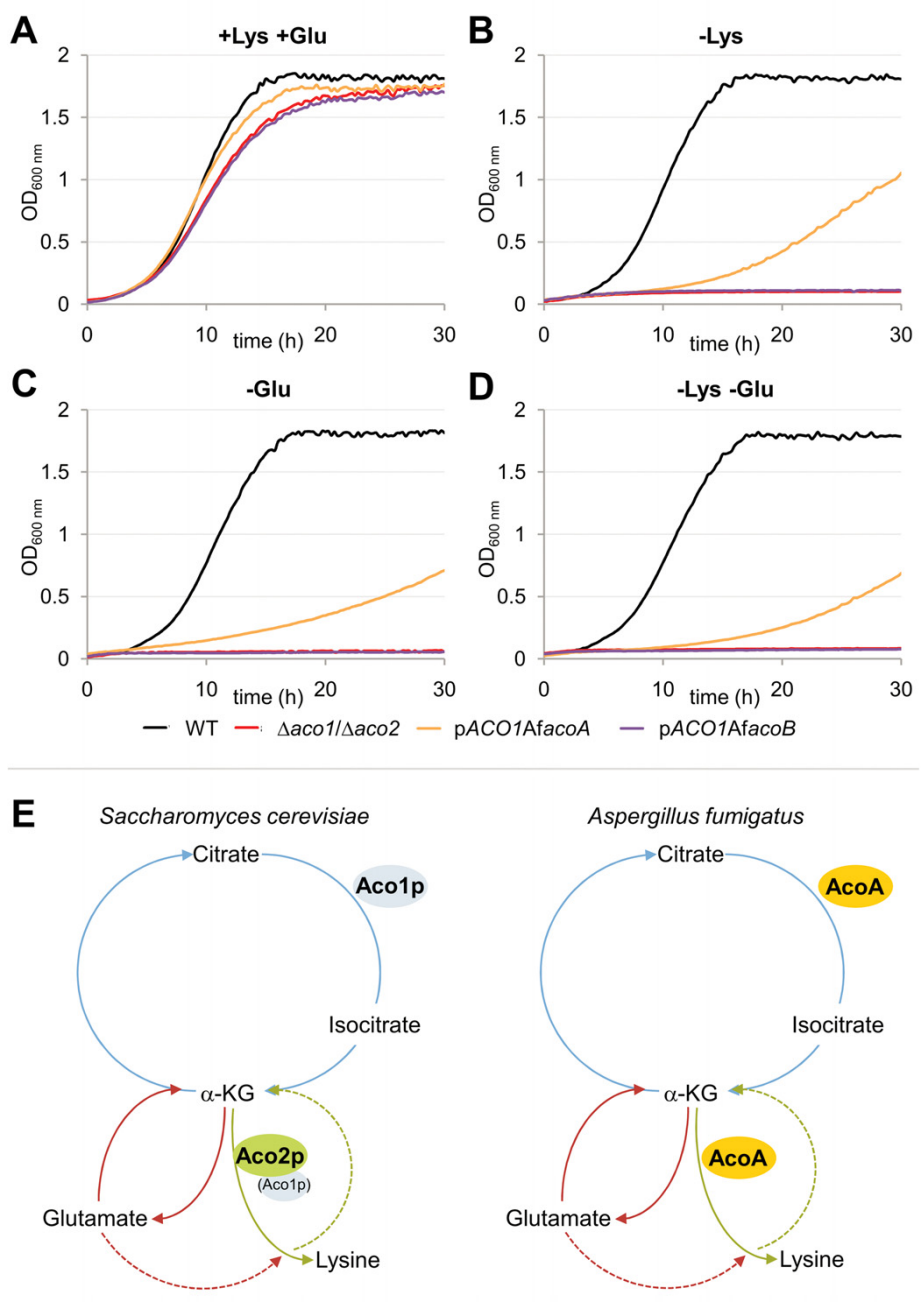
Complementation analysis of the *S. cerevisiae* Δ*aco1*/Δ*aco2* mutant with *A. fumigatus* *acoA* and *acoB* (A–D) and scheme of the proposed roles of aconitases in *S. cerevisiae* and *A. fumigatus* (E). Complementations are depicted only for *A. fumigatus* genes under control of the *S. cerevisiae* *ACO1* promoter and results from liquid cultures are shown. WT = wild type, Δ*aco1*/Δ*aco2* = mutant deleted in the *aco1* and *aco2* gene, p*ACO1AfacoA* = Δ*aco1*/Δ*aco2* mutant complemented with the *A. fumigatus* *acoA* gene, p*ACO1AfacoB* = Δ*aco1*/Δ*aco2* mutant complemented with the *A. fumigatus* *acoB* gene. For complete complementation analyses on solid media, refer to Fig. S3. A. With lysine and glutamate (+Lys +Glu) all strains grow at similar rates. B. When lysine is omitted (−Lys), *A. fumigatus* AcoA, but not AcoB, partially complements lysine auxotrophy. C. When glutamate is omitted, a partial complementation is also observed with AcoA, but not with AcoB. D. The same is true for media lacking both amino acids (−Lys −Glu). This leads to the schemes in E, in which we conclude that *S. cerevisiae* separates contribution to glutamate and lysine biosynthesis on two enzymes, i.e. Aco1p mainly for glutamate and Aco2p for lysine biosynthesis, whereas a single citric acid cycle aconitase in *A. fumigatus* contributes to both syntheses.

As *A. fumigatus* AcoA is essential for growth even in the presence of lysine and glutamate, we were unable to dissect the specific contribution of this enzyme to the respective amino acid biosyntheses. Therefore, we performed a complementation approach in the *S. cerevisiae aco1*/*aco2* double deletion mutant. The *acoA* and *acoB* genes from *A. fumigatus* were fused with the mitochondrial import sequence and the promoter of either *S. cerevisiae ACO1* or *S. cerevisiae ACO2*, cloned in the pYES vector and used for transformation of the *S. cerevisiae aco1*/*aco2* double mutant. Growth of the transformants on YPD or complete glucose medium remained without obvious phenotype (Figs 7 and S3). However, the *acoA* gene, but not the *acoB* gene, partially complemented the glutamate and lysine auxotrophic phenotype of the *aco1*/*aco2* double mutant strain regardless of the use of the *ACO1* or *ACO2* promoter for driving gene expression (Figs 7 and S3). Similarly, when *A. nidulans acoA* and *acoB* were cloned under control of the *ACO1* promoter, a weak but significant complementation was only observed with the *acoA* gene (Fig. S3). These results again confirm that AcoB does not contribute to amino acid biosynthesis, while AcoA from *Aspergillus* species seems to support the citric acid cycle and amino acid synthesis under *in vivo* conditions. Thus, while *S. cerevisiae* separates glutamate synthesis via the citric acid cycle and lysine biosynthesis via the α-aminoadipate pathway on two separate aconitases, data imply that a single constitutively active AcoA protein is solely responsible for these functions in Aspergilli (Fig. 7E).

### Phylogeny of aconitases and homoaconitases from fungal, bacterial and archaeal origin

**Fig. 8:**
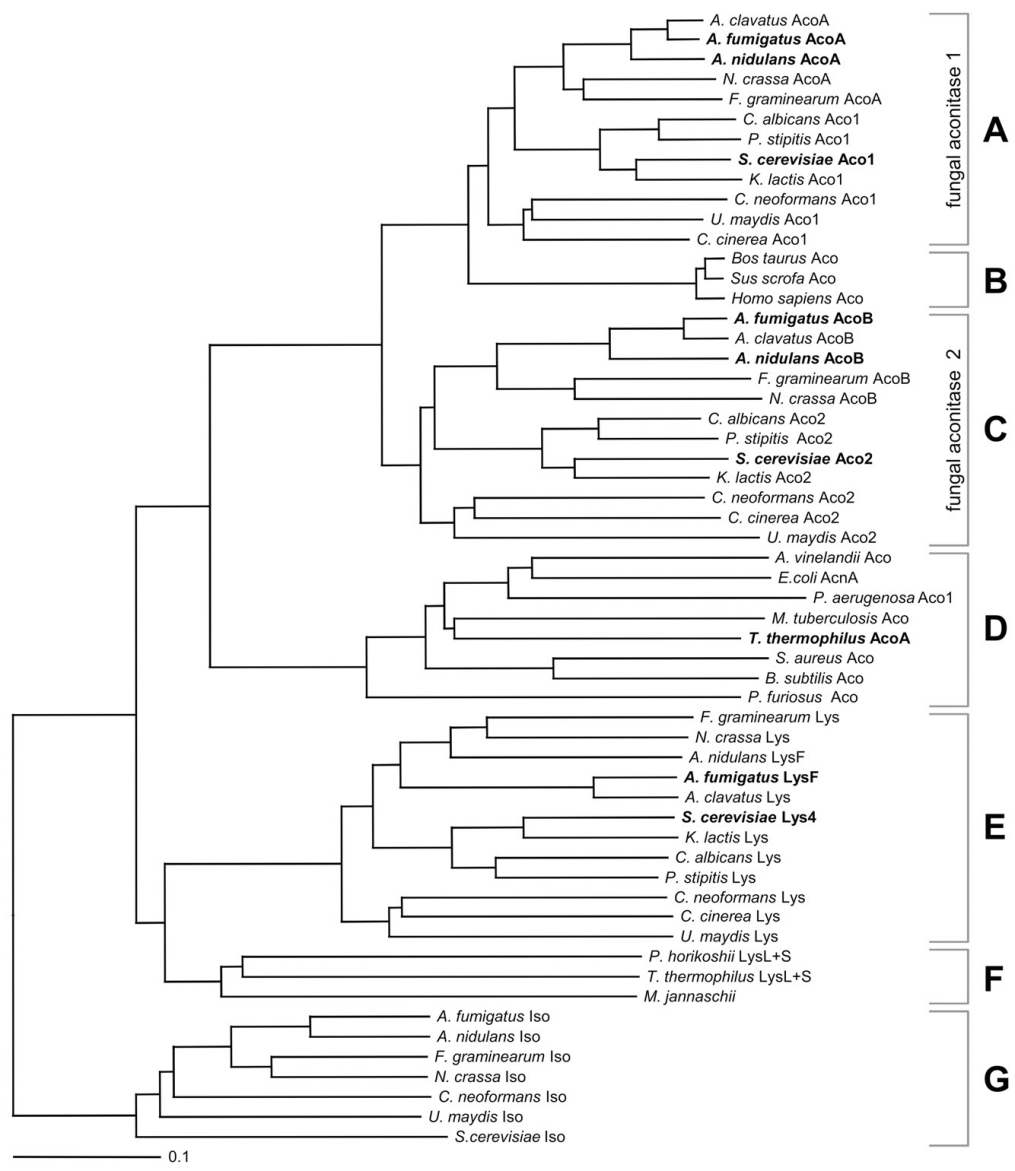
Phylogenetic analysis of aconitase family proteins. Sequences from fungal and bacterial aconitases, homoaconitases (mainly of fungal origin, except the proteins from the bacterium *T. thermophilus* and the archaea *Pyrococcus horikoshii* and *M. jannaschii*) and isopropylmalate dehydratases from fungi were used for tree construction. Enzymes purified and characterized in this study are marked in bold. Proteins can be divided in seven main groups: (A) fungal aconitase group 1, (B) mammalian aconitase, (C) fungal aconitase group 2, (D) bacterial aconitases, (E) fungal homoaconitase, (F) prokaryotic/archaea homoaconitase, (G) fungal isopropylmalate dehydratases. Accession number of proteins can be found in *Supporting information*. For tree construction isopropylmalate dehydratases served as out-group. Homoaconitases are only distantly related to aconitases. In contrast, fungal group 2 aconitases appear closely related to fungal group 1 and mammalian aconitases.

To obtain insights into the evolutionary relationships between isopropylmalate dehydratases, aconitases and homoaconitases from various origins (see also Fig. 1), a phylogenetic tree was constructed (Fig. 8). As already shown by others (Irvin and Bhattacharjee, 1998; Nishida *et al*., 1999), fungal homoaconitases appear to be only distantly related to fungal aconitases and isopropylmalate dehydratases. In general, isopropylmalate dehydratases, homoaconitases and aconitases formed three large distinct clades, whereby homoaconitases appeared more closely related to isopropylmalate dehydratases than to aconitases. Within each clade, archaeal and bacterial enzymes are separated from eukaryotic enzymes and occupy basal positions and the tree architecture more or less follows the usual taxonomic relationships. Thus, one might assume that an aconitase-like precursor in the eukaryotic lineage was duplicated. Subsequently, one enzyme specified for the citric acid cycle, whereas the second enzyme might have served as precursor for isopropylmalate dehydratases and homoaconitases in leucine and lysine biosynthesis respectively.

Within the aconitase clade, eukaryotic citric acid cycle aconitases and fungal aconitases of the *S. cerevisiae* Aco2p subfamily formed two distinct, but closely related clusters. This implies that an evolutionary more recent duplication event in fungi provided the origin for this second aconitase. The high substrate specificity of Aco2p for homoaconitate implies that specific mutations occurred that eased positioning of homocitrate in the ‘citrate mode’ within the active site of the enzyme. Interestingly, the lack of activity of AcoB proteins from *Aspergillus* species suggests that these enzymes either never gained this new function or lost it due to the essential presence and activity of the citric acid cycle aconitase. Alternatively, AcoB proteins from filamentous fungi may have adapted to other yet unknown substrates.

### Role of an active site lysine in Aco2p substrate specificity

As deduced from phylogenetic analyses, we assumed that site-specific mutations could have altered the substrate specificity of *S. cerevisiae* Aco2p for accepting homoaconitate rather than aconitate as a substrate. To determine specific amino acids in Aco2p that might be responsible for the altered substrate specificity, we compared conserved active site residues essential for catalysis in citric acid cycle aconitases with those present in Aco2p-like proteins.

Virtually all amino acids that had been described to be essential for substrate orientation and catalytic activity in eukaryotic aconitases (Lauble *et al*., 1992; Zheng *et al*., 1992; Beinert *et al*., 1996) were conserved in all proteins of the Aco2p subclade including the catalytically inactive AcoB proteins from *Aspergillus* species. However, a single exception derived from a lysine at position 610 in the native sequence of Aco2p that consists of an arginine in citric acid cycle aconitases (R604 in Aco1p). In aconitases this arginine has been assumed to fix substrates in the correct position for catalysis (Zheng *et al*., 1992). Thus, we speculated that a mutation of arginine to lysine could be essentially involved in changes of substrate specificity. To confirm this assumption we performed site-directed mutations in Aco1p (R604K) and Aco2p (K610R) and purified the recombinant proteins (Fig. S1C and D). After reconstitution of the iron–sulphur cluster, enzymatic activities were determined with aconitate and homoaconitate as substrates (Table 2).

**Table 2:** Activities of mutated recombinant *S. cerevisiae* aconitases Aco1pR604K and Aco2pK610R in comparison to wild-type enzymes

Enzyme	A_spec_ aconitate (U mg^−1^)	Rel. activity to wild-type enzyme^a^	A_spec_ homoaconitate (U mg^−1^)	Rel. activity to wild-type enzyme^b^	Ratio A_spec_ aconitate : homoaconitate
Aco1pR604K	0.0104	0.4%	0.0135	1.5%	1:1.3
Aco2pK610R	0.575	11 500%	2.66	55.9%	1:4.6

a Refers to data from Table 1: Aco1p with aconitate 23.7 U mg^−1^; Aco2p with aconitate 0.005 U mg^−1^.

b Refers to data from Table 1: Aco1p with homoaconitate 0.9 U mg^−1^; Aco2p with homoaconitate 4.76 U mg^−1^.

The arginine to lysine mutation (R604K) in Aco1p strongly diminished activity with both substrates, aconitate and homoaconitate. This confirms the essential role of the arginine residue for substrate positioning in the active site of citric acid cycle aconitases. However, despite the low overall activity of mutated Aco1p, the maximum activity with homoaconitate was similar to that with aconitate, indicating that the arginine residue is more important for aconitate than for homoaconitate binding. This assumption was further supported by analysis of the mutated Aco2p protein. When the lysine in Aco2p was mutated to an arginine, this enzyme lost approximately 45% of its maximum activity with homoaconitate. However, activity with aconitate strongly increased from 0.005 U mg^−1^ to 0.58 U mg^−1^ (factor 116). Thus, the conversion of arginine to lysine diminishes citric acid cycle aconitase activity and explains why the Aco2p protein cannot restore glutamate prototrophy of *aco1* mutants. Additionally, this mutation in Aco2p may have released the enzyme from citric acid cycle responsibilities and allowed the evolutionary adaptation to specifically serve for the α-aminoadipate pathway. However, as the arginine to lysine exchange is also present in AcoB proteins from *Aspergillus* species, which are catalytically inactive with either substrate, the contribution of additional mutations in this adaptation process remains unclear.

## Discussion

In this study we elucidated the enzymatic isomerization of homocitrate to homoisocitrate, an essential step in the fungal α-aminoadipate pathway for lysine biosynthesis (Fig. 1). Previous investigations on deletion mutants revealed that homoaconitase essentially contributes to this reaction (Bhattacharjee, 1985; Weidner *et al*., 1997). However, it remained unclear, whether homoaconitases perform the complete isomerization reaction. By purifying recombinant fungal homoaconitases we were able to show that, similar to homoaconitase from the thermophilic bacterium *T. thermophilus* (Jia *et al*., 2006), this class of enzymes only catalyses the reversible reaction between homoaconitate and homoisocitrate, but not between homoaconitate and homocitrate. Thus, our study clearly indicates that two independent enzymes are involved in the isomerization. In subsequent experiments we demonstrated that aconitases are the second contributors to this reaction.

**Fig. 9:**
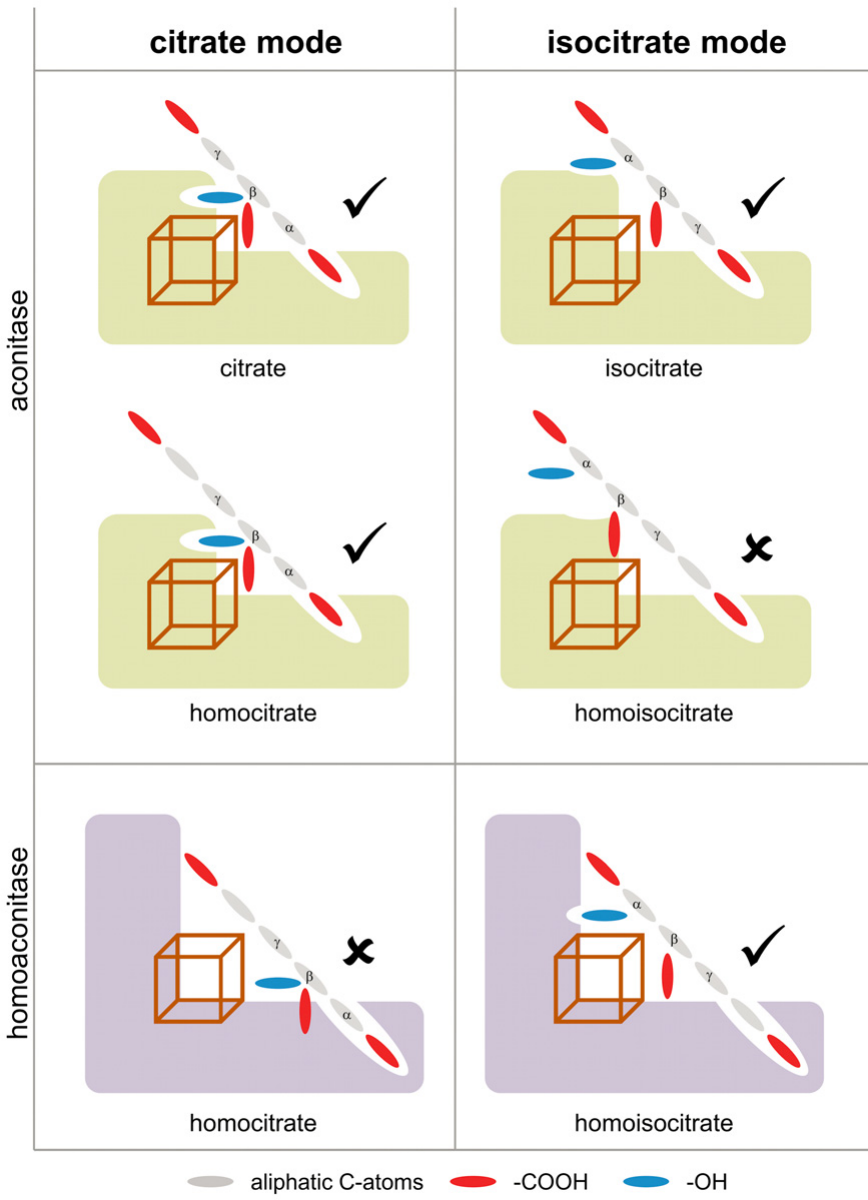
Schematic overview of substrate binding in the active site of aconitases and homoaconitases in citrate and isocitrate binding mode. Hooked pictures indicate confirmed binding, whereas an inability to bind is marked by a cross. α, β, γ indicate the C-atoms important for binding of the respective substrate in the active site. The brownish cube depicts the iron–sulphur cluster. Binding in the isocitrate mode requires a 180° rotation in relation to the citrate mode (see also Fig. 1). Aconitases are able to bind homocitrate in the citrate mode for water abstraction. In contrast, the enlarged aliphatic chain of homoisocitrate prohibits correct substrate positioning in the isocitrate mode. Similarly, homoaconitases are specifically adapted for correct orientation of homoisocitrate, but cannot simultaneously bind homocitrate.

Our experiments confirm that homoaconitases essentially require an iron–sulphur cluster for catalytic activity, as all recombinant enzymes were purified as inactive apoenzymes but regained their activity after *in vitro* reconstitution of the iron–sulphur cluster. Thus, in principle, homoaconitases follow the same reaction mechanism as described for aconitases (Rose and O'Connell, 1967; Lauble and Stout, 1995). However, citric acid cycle aconitases accomplish the complete isomerization from citrate to isocitrate via the intermediate aconitate (Lauble *et al*., 1992; 1994; Lauble and Stout, 1995). Aconitases can bind aconitate in two different orientations, which is resembled by a 180° ‘flip’ of the substrate leading to either citrate or isocitrate formation during the *trans*-addition of water (Lauble and Stout, 1995). To allow for this alternate binding, aconitase needs to undergo a conformational change of the active site to harbour the C_γ_-acetyl side-chain in respect to the binding mode of aconitate. Thus, two questions need to be addressed: (i) when following a similar reaction mechanism, why do homoaconitases only perform a single step of the isomerization reaction, i.e. the reversible reaction between homoaconitate and homoisocitrate? (ii) As our analyses have shown that aconitases are involved in the isomerization reaction, why do these enzymes only catalyse the reaction resembling binding of homoaconitate in the citrate mode? A possible explanation is depicted in the schematic drawing of Fig. 9.

Assuming that homocitrate binds in the citrate mode to aconitases, the active site of the enzyme needs to position the C_α_ and C_β_ atoms of homocitrate correctly to the iron–sulphur cluster to allow for the *trans*-elimination of water. This also means that in the citrate binding mode, the C_γ_-propionyl side-chain of homocitrate must find sufficient space within the active site to allow the correct positioning of the C_α_ and C_β_ atoms. In contrast, binding of the ‘flipped’ homoaconitate formed after water abstraction may be hindered by the molecular size of the C_γ_-propionyl side-chain, which seems to occupy too much space for correct positioning of the C_α_ and C_β_ atoms of homoaconitate at the iron–sulphur cluster. Thus, the additional space for harbouring the C_γ_-propionyl side-chain in the isocitrate mode seems to be realized in homoaconitases, but is paid at the cost of correct positioning of homoaconitate in the citrate mode. This structural adaptation of homoaconitases for homoaconitate binding in the isocitrate mode seems to require multiple amino acid exchanges that led to the phylogenetic separation of homoaconitases from aconitases (Fig. 8). The hypothesis on the separation of the de- and rehydration reactions on two enzymes due to structural interferences is also supported by studies on the methylcitrate cycle required for propionate degradation. Here, the intermediate (2*S*,3*S*)-methylcitrate requires a *syn*-elimination of water to form methyl-*cis*-aconitate (Brock *et al*., 2002). As aconitases can only perform *trans*-eliminations of water, this reaction requires a specific methylcitrate dehydratase. Additionally, binding of methylcitrate in the ‘citrate mode’ is sterically hindered by interference of the methyl-group with an essential aspartate residue (Lauble and Stout, 1995). However, methyl-*cis*-aconitate can bind in the ‘isocitrate mode’ in aconitases, which then complete the formation of (2*R*,3*S*)-methylisocitrate in the methylcitrate cycle by *trans*-addition of water (Brock *et al*., 2002).

In terms of the aconitase contribution to lysine biosynthesis, our study additionally points to a difference between yeasts and filamentous fungi. Despite the existence of an Aco2p-like protein in filamentous fungi (Fig. 8), the citric acid cycle aconitase seems to be responsible for homocitrate dehydration. This assumption derives from the observation that recombinant AcoB proteins from *A. fumigatus* and *A. nidulans* (i) showed no activity in *in vitro* assays, (ii) did not complement the *S. cerevisiae* aconitase double mutant and (iii) the deletion of *acoB* in *A. fumigatus* provoked no growth defect or increased lysine requirement. However, to draw a general conclusion on Aco2p-like proteins in filamentous fungi, homologous enzymes from other species need to be investigated in detail.

In contrast, in the yeast *S. cerevisiae* the aconitase Aco2p specifically contributes to lysine biosynthesis as deduced from the observations that: (i) Aco2p only plays a negligible role in the citric acid cycle, as the glutamate auxotrophy of an *aco1* mutant is not complemented by Aco2p, (ii) an *aco2* mutant shows a stronger dependence on lysine supplementation than an *aco1* mutant to reach wild-type growth rates and (iii) the purified recombinant Aco2p displays very low activity with aconitate, but is highly active with homoaconitate. This adaptation to homoaconitate seems to derive from an arginine to lysine exchange in the active site, as reversal of this mutation in Aco2p reduced homoaconitate substrate specificity. In contrast, the arginine to lysine mutation in Aco1p produced an aconitase with strongly diminished aconitase activity. The phylogenetic relationship between Aco1p and Aco2p implies that one enzyme derived from a gene duplication event with subsequent specification for the new substrate and metabolic pathway. This process is similar to the evolution of fungal methylisocitrate lyases from isocitrate lyases and seems to depict a general mechanism in fungi to enhance and adapt their metabolic potential (Müller *et al*., 2011).

Nevertheless, one might ask why the yeast *S. cerevisiae* contains such a second active aconitase that specifically serves for the α-aminoadipate pathway, whereas this is not the case for the second aconitase from filamentous fungi. The reason might derive from significant differences in the metabolic physiology of yeasts and filamentous fungi. Yeasts generally tend to fermentation accompanied by overflow metabolism when grown in the presence of glucose. Thus, glucose is mainly converted to ethanol, acetate and glycerol without further metabolism via the citric acid cycle (Fleck and Brock, 2008; Heyland *et al*., 2009). Although the absence of glutamate from glucose medium leads to strong transcriptional activation of *ACO1*, external addition of glutamate significantly reduces *ACO1* expression and Aco1p is no longer essentially required to support growth. This is also reflected by our growth analyses on complete media on which no striking growth defect was recorded for the *aco1* mutant. Thus, under specific growth conditions the citric acid cycle is dispensable, which is confirmed by the viability of different *S. cerevisiae* citric acid cycle mutants (Lin *et al*., 2008). As Aco2p does not contribute to the citric acid cycle, it cannot recover glutamate auxotrophy derived from *aco1* deletion. However, due to its independent function Aco2p can contribute to lysine biosynthesis in the absence of citric acid cycle activity. Nevertheless, the presence of glutamate remains essential, as α-ketoglutarate is required for the initial formation of homocitrate. Additionally, glutamate is a donor of amino groups in the subsequent transamination reactions of the α-aminoadipate pathway.

In contrast to yeasts, filamentous fungi seem to essentially require the citric acid cycle for energy production and, thus, the citric acid cycle is constitutively active. In our study, several attempts to generate an *acoA* deletion mutant in *A. fumigatus* failed, but we successfully replaced the original *acoA* promoter by the xylose inducible *xylP* promoter (Zadra *et al*., 2000). The resulting strain was only able to grow under inducing conditions regardless of the presence or absence of glutamate or lysine. Thus, the essential contribution of AcoA for growth prohibits the assessment of the specific contribution of AcoA to amino acid biosynthesis. However, in contrast to *S. cerevisiae* Aco1p, which is highly specific for citric acid cycle substrates with a ratio of 26:1 between aconitate and homoaconitate conversion, this ratio is much more balanced (approximately 6:1) in citric acid cycle aconitases from *A. fumigatus*, *A. nidulans* and also *T. thermophilus* (Table 1). Therefore, the citric acid cycle aconitase in filamentous fungi and from *T. thermophilus* seems to possess sufficient capacity to simultaneously deal with its function in the citric acid cycle and the α-aminoadipate pathway. In this respect it is worth to note that the identification of a functional aconitase from *T. thermophilus* confirms previous assumptions that an aconitase could also be involved in the bacterial isomerization of homocitrate into homoisocitrate (Jia *et al*., 2006).

However, two questions need to be addressed in future analyses. (i) Do fungi with extremely high penicillin production levels, such as *P. chrysogenum*, contain a second aconitase specifically adapted to the α-aminoadipate pathway that satisfies the high demand of α-aminoadipate? In this respect, an AcoB homologue is present in the genome of *P. chrysogenum* (accession: CAP91380) that shows 86% identity to *A. fumigatus* AcoB and 59% to *S. cerevisiae* Aco2p. However, its biochemical characteristics have not been studied yet. (ii) Has the second aconitase in Aspergilli and other filamentous fungi never obtained the specificity to serve for the α-aminoadipate pathway or was this activity subsequently lost due to the sufficing activity of the citric acid cycle aconitase?

In conclusion, we were able to provide evidence for a two-step isomerization reaction of homocitrate to homoisocitrate in the α-aminoadipate pathway involving an aconitase and the homoaconitase. The knowledge on the interaction of citric acid cycle aconitases with lysine biosynthesis could be important for flux calculations during the optimization process of penicillin and lysine production from fungi. Especially for penicillin production the detailed characterization of the second aconitase in production strains appears important. As α-aminoadipate formation is a rate-limiting step in penicillin production (Jaklitsch *et al*., 1986; Casqueiro *et al*., 1999), the introduction of a highly expressed synthetic codon-adapted version of *S. cerevisiae* Aco2p could possibly enhance production rates.

## Experimental procedures

### Strains and growth conditions

All cultivations of *S. cerevisiae* wild type and mutant strains were performed at 30°C. All *S. cerevisiae* strains used in this study are listed in Table S2. *S. cerevisiae* strains were cultivated either in YPD or in complete minimal media with glucose as carbon source (Fleck and Brock, 2009). When required, media were supplemented with 0.2 mM uracil, 0.3 mM histidine, 1–2 mM glutamate or 1–2 mM lysine. In plate assays media were solidified by the addition of 1.5–2% (w/v) agar prior to sterilization. When the *GAL1* promoter was used for gene expression, glucose was replaced by 2% (w/v) galactose. For growth curves in liquid media and spot dilution assays on solid media, *S. cerevisiae* strains were pre-cultured for 12 h in YPD medium. Cells were harvested by centrifugation, washed four times in H_2_O and optical density was measured in an UV/Vis Lambda 25 double beam spectrophotometer (Perkin Elmer, Rodgau, Germany) or a FLUOstar Omega (BMG Labtech, Jena, Germany) at 600 nm. When growth curves were recorded from 50 ml of shake flask cultures, media were inoculated with an initial optical density at OD_600_ of 0.1 and cultures were incubated on a rotary shaker at 200 r.p.m. Aliquots were removed at distinct time points for OD_600_ determinations. Additionally, growth curves for all yeast strains were recorded in a microtitre plate format. Transparent flat-bottom 96-well plates (Nunc, Thermo Scientific GmbH, Schwerte, Germany) contained 200 μl of the respective growth medium and yeast cells at a starting OD_600_ of 0.02. Plates were sealed with sterile transparent adhesive sealing film (Sigma-Aldrich GmbH, Taufkirchen, Germany) and incubated inside the FLUOstar Omega microplate reader at a constant temperature of 30°C. Plates were shaken every 15 min prior to measurement of the OD_600_ and incubation was continued for a total time of 30 h. Blank values were subtracted from all data points and growth curves were generated using the Microsoft Excel software.

*Aspergillus fumigatus* strain CBS 144.89 (Centraalbureau voor Schimmelcultures, Utrecht, the Netherlands) was the parental strain in all experiments. For gene deletions a derivative with deleted *akuB* gene was used that shows increased frequency of homologous recombination (da Silva Ferreira *et al*., 2006). *A. fumigatus* was cultivated at 37°C and liquid cultures were shaken at 200 r.p.m. Growth media were based on the *Aspergillus* minimal medium (AMM) (http://www.fgsc.net/methods/anidmed.html) with addition of 50 mM glucose or 100 mM ethanol. When required, 5 mM lysine was added to growth media. Media were inoculated with conidia suspensions. These suspensions derived from cultures grown for at least 48 h at 37°C on AMM glucose agar plates. Conidia were harvested in sterile water and hyphal elements and clumps were removed by filtration over a 40 μm cell strainer (BD, Heidelberg, Germany). *A. nidulans* strain FGSCA4 (Fungal genetic stock centre, Kansas, USA) served for isolation of genomic DNA and RNA and was cultivated identical to *A. fumigatus* in AMM with glucose. The *A. fumigatus* transformant expressing the aconitase *acoA* under control of the *xylP* promoter from *P. chrysogenum* was maintained on 50 mM xylose containing minimal medium. Carbon shift experiments were performed by incubating *A. fumigatus* strains for 24 h at 37°C and 200 r.p.m. shaking on 50 mM xylose liquid medium. Subsequently, mycelium was harvested over sterile miracloth (Merck/Calbiochem, Schwalbach/Ts. Germany) extensively washed with phosphate-buffered saline and transferred to fresh glucose or xylose-containing media.

For DNA isolation from *T. thermophilus* STI11057 (HKI strain collection), the strain was grown in liquid medium composed of yeast extract (4 g l^−1^), peptone (8 g l^−1^) and sodium chloride (2 g l^−1^). Cultures were incubated at 60°C and 200 r.p.m. on a rotary shaker.

*Escherichia coli* strain DH5α (Invitrogen GmbH) was used for amplification of plasmids and was cultivated on Luria–Bertani medium. For recombinant protein production *E. coli* BL21 (DE3) Rosetta 2 cells (Novagen, Darmstadt, Germany) were used, which were incubated at 18–30°C on a rotary shaker at 200 r.p.m. in Overnight express Instant TB medium (Novagen). When required, media were supplemented with ampicillin (final concentration: 0.1 mg ml^−1^) and chloramphenicol (final concentration: 34 μg ml^−1^).

### Isolation of plasmid DNA, genomic DNA, total RNA and generation of cDNA

Genomic DNA (gDNA) was isolated from yeasts and filamentous fungi by using the MasterPure Yeast DNA Purification Kit (Epicentre, Madison, WI, USA) as recommended by the manufacturer. For filamentous fungi the mycelium was ground under liquid nitrogen to a fine powder prior to DNA extraction. Small-scale plasmid purifications from *E. coli* or *S. cerevisiae* were performed using the NucleoSpin Plasmid Kit (Machery-Nagel GmbH & Co. KG, Düren, Germany). For larger scales the NucleoBond Xtra Midi Kit (Machery-Nagel) was used. Total RNA was isolated from powdered mycelium of filamentous fungi by using the RiboPure-Yeast Kit (Ambion/Applied Biosystems, Darmstadt, Germany) as recommended by the manufacturer. From *S. cerevisiae* total RNA was isolated with the MasterPure Yeast RNA Purification Kit (Epicentre). If not indicated otherwise, cDNA was generated from total RNA using the SuperScript III reverse transcriptase (Invitrogen GmbH) with anchored oligo-dT primers.

### Recombinant production of aconitases, homoaconitases and homoisocitrate dehydrogenases

If not indicated otherwise, the following procedure was used to generate expression constructs for recombinant protein production in *E. coli*. Genes were either amplified from genomic DNA or cDNA in dependence of intron sequences interrupting the open reading frame. Genes were amplified with the high-fidelity Phusion polymerase (Finnzymes/Thermo Scientific GmbH) and the gene-specific oligonucleotides listed in Table S3. Oligonucleotides additionally contained specific restriction sites that served for downstream cloning procedures. PCR products were purified from agarose gels and cloned into the pJET1.2 vector of the CloneJet PCR Cloning Kit (Fermentas/Thermo Scientific GmbH). The following templates and oligonucleotide pairs were used for gene amplifications: *S. cerevisiae ACO1* = gDNA with P1 and P2; *S. cerevisiae ACO2* = gDNA with P3 and P4; *A. fumigatus acoA* = cDNA with P5 and P6; *A. fumigatus acoB* = gDNA with P7 and P8; *A. nidulans acoA* = cDNA with P9 and P10; *A. nidulans acoB* = gDNA with P11 and P12; *T. thermophilus acoA* = gDNA with P13 and P14; *S. cerevisiae* homoaconitase *LYS4* = gDNA with P15 and P16; *S. cerevisiae* homoisocitrate dehydrogenase *LYS12* = gDNA with P17 and P18. The homoaconitase *lysF* from *A. fumigatus* was amplified and cloned by a slightly different procedure. Total RNA was used as a template in first strand gene-specific reverse transcription PCR with Bioscript reverse transcriptase (Bioline GmbH, Luckenwalde, Germany) in the presence of oligonucleotides P19 and P20. Subsequently, the polymerase Immolase (Bioline GmbH) was added and the first strand cDNA was amplified. The resulting PCR product was gel-purified and cloned into the pCRII TOPO vector (Invitrogen GmbH). Subsequent procedures were identical for all genes. All genes were excised from their initial cloning vector by using the restriction sites indicated in the list of oligonucleotides (Table S3). All genes were subcloned into a modified pET43.1H6 vector (Hortschansky *et al*., 2007) allowing the production of proteins with N-terminal His-tag. As gene expression was controlled by a T7 promoter, all expression plasmids were transferred to *E. coli* BL21 (DE3) Rosetta 2 cells.

### Cloning procedures for generation of *A. fumigatus* and *S. cerevisiae* deletion mutants and complementation constructs

Detailed descriptions for the following procedures can be found in *Supporting information*: generation of *A. fumigatus* homoisocitrate dehydrogenase and aconitase B deletion mutants; complementation of *S. cerevisiae lys12* mutant with the *A. fumigatus lysB* gene; generation of a *S. cerevisiae aco1*/*aco2* double deletion mutant; generation of the 2 μm plasmid pYES_HIS3; complementation of the *S. cerevisiae aco1*/*aco2* double deletion mutant with *Aspergillus* aconitase genes; complementation of *aco1*/*aco2* deletion mutant with *S. cerevisiae ACO1* and *ACO2*. Genotypes of resulting strains and all oligonucleotides used in the cloning procedures are listed in Tables S2 and S3 respectively.

### In locus replacement of the *A. fumigatus* *acoA* promoter by the *xylP* promoter from *P. chrysogenum*

A detailed description for the replacement procedure of the *A. fumigatus acoA* promoter by the *P. chrysogenum xylP* promoter is provided in *Supporting information*. In brief, a construct was generated that contained a fusion of the following fragments in the given order: (i) an upstream *acoA* promoter fragment, (ii) the pyrithiamine resistance cassette *ptrA*, (iii) the xylose inducible *xylP* promoter from *P. chrysogenum* fused with (iv) the 5′ coding region of the *acoA* gene. The construct was flanked by HindIII restriction sites that allowed excision from the cloning vector. Protoplasts of the *A. fumigatus* Δ*akuB* strain were transformed with the construct and regenerated on xylose and pyrithiamine-containing media. Transformants were checked by PCR and Southern blot analysis for correct promoter replacement.

### Site-directed mutagenesis of *S. cerevisiae* *ACO1* and *ACO2*

To replace arginine 604 by lysine in Aco1p and lysine 610 by arginine in Aco2p, site-directed mutagenesis was performed by fragment annealing PCR. For both genes, complementary oligonucleotides were deduced that carried the desired mutation. For mutation of *ACO1* and *ACO2*, PCR was performed with Phusion polymerase using the respective pET expression vector as template. For *ACO1* the region upstream of the mutation was amplified with P1 and P75 and the downstream region with P2 and P76. Similarly, for *ACO2* the pairs P3 and P77 for the upstream and P4 and P78 for the downstream region were used. All PCR fragments were gel-purified and 0.5 μl aliquots of the respective upstream and downstream fragment were mixed. These fragments were denatured, annealed and elongated by Phusion polymerase. Subsequently, oligonucleotides P1 and P2 for *ACO1* and P3 and P4 for *ACO2* were added and mutated genes were amplified by 33 cycles. After gel purification, genes were first cloned into pJET1.2, excised and subcloned into the pET expression vector as described above. Mutations were checked by sequencing of the complete *ACO1* and *ACO2* genes. For gene expression, plasmids were transferred to BL21 (DE3) Rosetta 2 cells and recombinant production and protein purification was performed as described below.

### Quantitative real-time PCR

Quantitative real-time PCR experiments were performed as described previously (Schöbel *et al*., 2010). In brief, *A. fumigatus* CBS144.89 was grown for 15 h in AMM with 50 mM glucose, AMM with 50 mM glucose and 5 mM lysine and AMM with 100 mM ethanol. RNA was isolated either by using the TRIsure Isolation Kit (Bioline GmbH) or by the MasterPure Yeast RNA Purification Kit (Biozym Scientific GmbH) as recommended by the respective manufacturer. *S. cerevisiae* CLIB334 was pre-cultured overnight in YPD medium, washed with water, transferred for 1 h to either YPD medium, complete medium without lysine and glutamate, complete medium (CM) without lysine, CM without glutamate and CM with 2 mM lysine and 2 mM glutamate. Tubulin served as standard gene for normalization of gene expression. Quantitative real-time PCR was performed on a StepOne Real-Time PCR system using the GeneAmp fast PCR kit (both Applied Biosystems) as described in the manufacturer's protocol and data were evaluated with the StepOne Real-Time PCR software package. EvaGreen dye (VWR International GmbH, Dresden, Germany) was used to visualize gene amplification. Oligonucleotides used for amplifications are listed in Table S3. Statistical significance was analysed with the paired *t*-test.

### Partial purification of the main aconitase from *A. fumigatus*

For partial purification of the main aconitase from *A. fumigatus*, strain CBS144.89 was cultured for 23 h in AMM-G50 medium. Mycelium was harvested over filter gaze (Miracloth, Calbiochem/Merck KGAA, Darmstadt, Germany), washed and pressed dry between tissue papers. Sixteen grams of mycelium was ground under liquid nitrogen and resuspended in 30 ml of buffer A (50 mM Tris/HCl pH 8; 1 mM citrate). Cell debris was removed by centrifugation at 31 000 *g* and subjected to ammonium–sulphate precipitation (first phase 0–50%, second phase 50–80%). The pellet from 50–80% saturation was resuspended in buffer B (50 mM Tris/HCl pH 8; 1 mM citrate; 1 M (NH4)_2_SO_4_) and loaded on a Phenylsepharose column (20 ml bed volume). Proteins were eluted by a gradient from 100% buffer B to 100% buffer A. Active fractions were combined, concentrated on centrifugal filter devices and desalted against buffer A on a NAP5 column (GE Healthcare, Munich, Germany). A Resource Q column (1 ml bed volume) was loaded and proteins were eluted by sodium chloride gradient from buffer A to buffer C (50 mM Tris/HCl pH 8; 1 mM citrate; 0.5 M NaCl). Due to overloading of the column, the flow-trough from the first run was concentrated and again applied to the washed and equilibrated Resource Q column. Active fractions were combined, concentrated and desalted against buffer A as described above. A 1 ml fraction was loaded on Reactive Red agarose column (2.7 ml bed size) and eluted by a sodium chloride gradient from buffer A to buffer D (50 mM Tris/HCl pH 8; 1 mM citrate; 1.5 M NaCl). Active fractions were combined, concentrated and analysed by SDS-PAGE. Bands were excised from the gel and subjected to a tryptic digest for MALDI-TOF MS-MS analysis as described previously (Brock *et al*., 2008).

### Purification of recombinant enzymes

Protein production was performed in 20–50 ml liquid Overnight express Instant TB medium. To identify growth conditions that produced a minimum of inclusion bodies as tested by SDS-PAGE analyses, incubations were performed in a temperature range between 18 and 30°C. When cells reached an optical density at 550 nm between 25 and 30, cells were collected by centrifugation, resuspended in 50 mM Tris/HCl pH 8.0 with 150 mM NaCl (buffer A) and disrupted three times for 2 min by sonication at 50% pulse and 50% intensity (Sonoplus; Bandelin electronic GmbH & Co. KG, Germany). Cell debris was removed by centrifugation at 30 000 *g* and filtered through a 0.45 μm filter. The supernatant was loaded onto a Ni-chelate column (bed size of 14 ml; GE Healthcare) using an ÄKTA Explorer system (GE Healthcare). After a stringency wash with buffer A + 20 mM imidazole, purified protein was eluted in buffer A containing 200 mM imidazole. Proteins were desalted by repeated concentration using centrifugal filter devices with a molecular cut-off of 30 kDa (Millipore). Purity of enzyme preparations was checked by SDS-gel electrophoresis on NuPage 4–12% Bis-Tris gels in a MES-buffered running system (Invitrogen GmbH). When required, an additional purification step using an anionic exchange ResourceQ column (1 ml bed size) was performed. Desalted proteins were loaded onto the column and elution was performed in Tris/HCl buffer pH 8.0 by applying a sodium chloride gradient from 0 to 0.5 M. Concentrated fractions were shock-frozen in liquid nitrogen and thawed on ice prior to introduction of iron–sulphur clusters.

### Reactivation of enzymatic activity

For reactivation of iron–sulphur cluster-containing enzymes, a modification of the protocol described for SufU reactivation (Albrecht *et al*., 2010) was followed. In brief, thiol-groups of purified enzymes were reduced by the addition of 2 mM fresh DTT and transferred to an anaerobic chamber (Hypoxystation H85; Meintrup DWS Laborgeräte GmbH, Holte, Germany). After desalting on Nap5 or Nap10 columns (GE Healthcare) against buffer (50 mM Tris/HCl pH 8, 150 mM NaCl, 1 mM DTT), the protein concentration was determined with Bradford reagent (Bio-Rad Laboratories, Munich, Germany). Protein concentrations were adjusted to a maximum of 3 mg ml^−1^ and DTT was added to a final concentration of 10 mM. After incubation for 1 h on ice Fe-ammonium citrate was added to a final concentration of 0.3 mM and the mixture was incubated for 5 min at room temperature. Li_2_S was added to a final concentration of 0.3 mM and incubation was continued for additional 3 h at room temperature. Unreacted iron and sulphide were removed by buffer exchange on a NAP10 column as described above. Aliquots of 50–200 μl were shock-frozen in liquid nitrogen.

### Activity determinations

Activity of aconitase family proteins was measured as described earlier (Fansler and Lowenstein, 1969). Homoisocitrate dehydrogenase activity was measured in a NAD^+^-dependent assay containing 50 mM Tris/HCl pH 8, 2.5 mM MgCl_2_, 0.2 mM NAD^+^ and 1 mM homoisocitrate. In a coupled assay homoisocitrate dehydrogenase was used as reporter enzyme to visualize the reactions from homocitrate to homoisocitrate. This coupled assay contained all compounds listed above with the exception of homoisocitrate. Homocitrate or homoaconitate were used as substrates and aconitases and/or homoaconitase were added in different combinations as indicated in *Results* section.

### Synthesis of substrates

Homoaconitic acid was kindly provided by D. Darley and W. Buckel (Marburg) and was produced by a method previously described by Massoudi *et al*. (1983).

#### Synthesis of homoisocitric acid

Homoisocitric acid was synthesized as described by Schmitz *et al*. (1996) and analytical data agreed with their results.

#### Synthesis of homocitric acid

Homocitric acid was obtained from homocitric acid lactone, which was synthesized by a procedure as previously described by Tavassoli *et al*. (2006) except that we used anion exchange chromatography for purification, which only yielded the lactone. In brief, a solution of sodium periodate (4 g, 18.7 mmol) in water (16 ml) was added drop wise to a suspension of silica gel (16 g) in dichloromethane (160 ml). Methyl (1*R*,3*R*,4*S*)-1,3,4-trihydroxycyclohexanecarboxylate (1.66 g, 8.73 mmol) in dichloromethane (30 ml) was added slowly. After 15 min, the starting material was consumed as confirmed by thin layer chromatography. The silica gel was collected on a glass filter and washed thoroughly with dichloromethane. The solvent was evaporated until crystallization occurred. Upon addition of formic acid (16 ml) and hydrogen peroxide (5 ml), the precipitate was dissolved and a clear brown solution formed. After 6 h of stirring, the formic acid was removed by rotary evaporation and 20 ml of potassium hydroxide (40%) was added to the residue. Saponification was complete within 1 h after which the mixture was neutralized with hydrochloric acid (1 M). The product was purified by anion exchange chromatography (Dowex 1 × 4, strong basic, 200–400 mesh, Sigma Aldrich). The column (25 cm × 5 cm) was washed with sodium formiate (1 M) until chloride ions could no longer be detected by a silver nitrate test. The column was then washed with eight column volumes of distilled water. The reaction mixture was applied to the column in a small volume of water. Fractions were eluted with water (3 × 400 ml) followed by 1 M formic acid (2 × 500 ml), 3 M formic acid (2 × 500 ml) and 6 M formic acid (6 × 400 ml). Homocitric acid was eluted with the 6 M formic acid fraction. Water and formic acid were removed by rotary evaporation followed by thorough drying under strong vacuum (< 10 Pa, 4 d) resulting in 517 mg (31.4%) of (-)-homocitric acid lactone; [α]D −55.2 °(c = 0.34, water); ^1^H NMR ((CD_3_)_2_CO) 3.20 (d, J = 17 Hz, 1 H), 3.01 (d, J = 17 Hz, 1 H), 2.4–2.7 (m, 4 H); ^13^C NMR ((CD_3_)_2_CO) 176.1, 172.2, 170.2, 83.2, 41.4, 31.6, 28.1. These analytical data agreed with previous results (Rodriguez and Biellmann, 1996).

To obtain homocitric acid from the lactone, a mixture of homocitric acid lactone (28 mg, 0.15 mmol), potassium hydroxide (10%, 1 ml) and methanol (0.1 ml) was stirred for 1.5 h and subsequently acidified with hydrochloric acid (1 M). A 20 μl sample was diluted in 500 μl of water. Analytical RP HPLC (Eurosphere 100, 5 μm, ambient temperature, 0.05% TFA in water, 1 ml min^−1^, isocratic) confirmed the identity of the product with a retention time of 10.7 min and a trace peak of homoaconitate.

### Phylogenetic analysis

The sets of aconitase and homoaconitase homologues in fungal and bacterial species were collected by applying Blast with AcoA (*A. fumigatus*). Archaeal and bacterial homoaconitases and aconitase were known from the literature (Nishida *et al*., 1999; Nishida, 2001; Jia *et al*., 2006; Drevland *et al*., 2008; van Vugt-Lussenburg *et al*., 2009). Accession numbers of all protein sequences are listed in Table S4.

The sequences were aligned with Muscle (Edgar, 2004). The phylogenetic analysis was performed using PhyML (Guindon and Gascuel, 2003) for the construction of the maximal likelihood tree and BioNJ at http://www.phylogeny.fr (Dereeper *et al*., 2008) for the construction of the neighbour-joining tree, with the Jones–Taylor–Thornton (JTT) model of the amino acid substitution in both cases. The NJ and ML trees had identical architecture.
