# Supplementary material for: The fungal α-aminoadipate pathway for lysine biosynthesis requires two enzymes of the aconitase family for the isomerization of homocitrate to homoisocitrate
Source: Mol Microbiol. 2012 Nov 6;86(6):1508–30. doi: 10.1111/mmi.12076 (PMC3556520; doi:10.1111/mmi.12076)
Supplement: Supplementary file 1 [file mmi0086-1508-SD1.pdf]

Supporting information

The fungal α-aminoadipate pathway for lysine biosynthesis requires two enzymes of the aconitase family for the isomerisation of homocitrate to homoisocitrate

Felicitas Fazius[1], Ekaterina Shelest[2], Peter Gebhardt[1,3] and Matthias Brock[1#]

[1]Microbial Biochemistry and Physiology, Leibniz Institute for Natural Product Research and Infection Biology, Hans-Knoell-Institute, Beutenbergstr. 11a, 07745 Jena, Germany

[2]Systems Biology/Bioinformatics, Leibniz Institute for Natural Product Research and Infection Biology, Hans-Knoell-Institute, Beutenbergstr. 11a, 07745 Jena, Germany

[3]Cell and Molecular Biology, Leibniz Institute for Natural Product Research and Infection Biology, Hans-Knoell-Institute, Beutenbergstr. 11a, 07745 Jena, Germany

[#]Corresponding author
Matthias Brock
Microbial Biochemistry and Physiology
Leibniz Institute for Natural Product Research and Infection Biology
Hans-Knoell-Institute
Beutenbergstr. 11a
07745 Jena, Germany

Tel.: ++49(0)3641 – 532 1710
E-mail: Matthias.brock@hki-jena.de

Figure S1: SDS-PAGE analysis of purified aconitases, homoaconitases and homoisocitrate dehydrogenase.

Figure S2: Growth analysis of *A. fumigatus* and *S. cerevisiae* homoisocitrate dehydrogenase mutants

Figure S3: Growth analysis of *S. cerevisiae* aconitase mutants and complemented strains on solid media in the presence and absence of lysine and glutamate.

Table S1: Half time of inactivation of iron-sulphur cluster containing aconitases and homoaconitases.

Table S2: *A. fumigatus* and *S. cerevisiae* strains used in this study.

Table S3: Oligonucleotides used in this study.

Table S4: Accession numbers of protein sequences used for phylogenentic analysis.

Detailed description of cloning procedures and strain constructions

Supplementary references

Figure S1: SDS-PAGE analysis of purified aconitases, homoaconitases and homoisocitrate dehydrogenase.

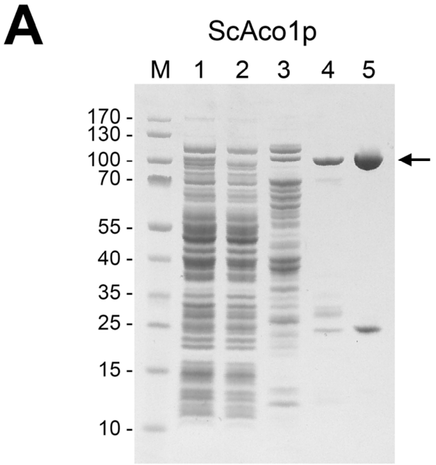

SDS-PAGE analysis of recombinant *S. cerevisiae* Aco1p purification: M, molecular mass marker: lane 1, cell-free extract (25 µg); lane 2, flow-through (20 µg); lane 3, stringency wash (12 µg); lane 4, elution fraction (2 µg); lane 5, Resource Q elution (approx. 8 µg)

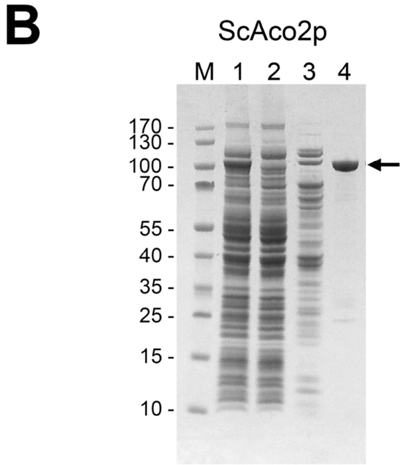

SDS-PAGE analysis of recombinant *S. cerevisiae* Aco2p purification: M, molecular mass marker: lane 1, cell-free extract (25 µg); lane 2, flow-through (25 µg); lane 3, stringency wash (10 µg); lane 4, elution fraction (2 µg)

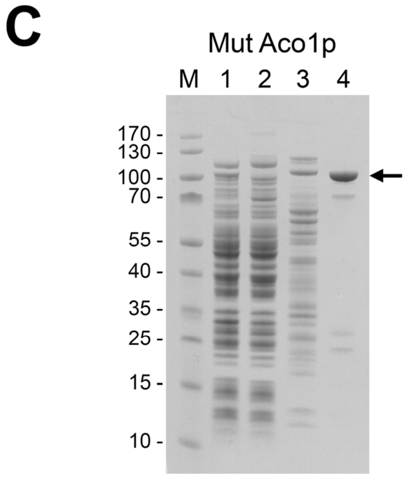

SDS-PAGE analysis of mutated recombinant *S. cerevisiae* Aco1p purification: M, molecular mass marker: lane 1, cell-free extract (25 µg); lane 2, flow-through (20 µg); lane 3, stringency wash (15 µg); lane 4, elution fraction (2 µg);

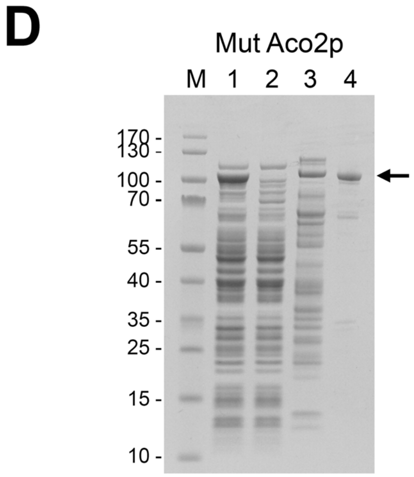

SDS-PAGE analysis of mutated recombinant *S. cerevisiae* Aco2p purification: M, molecular mass marker: lane 1, cell-free extract (25 µg); lane 2, flow-through (20 µg); lane 3, stringency wash (15 µg); lane 4, elution fraction (2 µg);

E

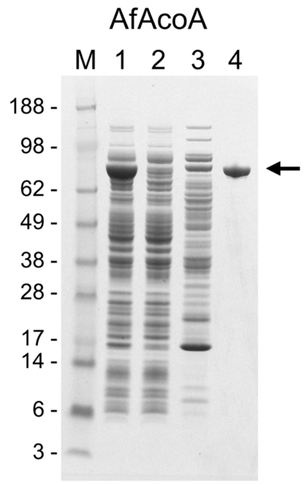

SDS-PAGE analysis of recombinant *A. fumigatus* AcoA purification: M, molecular mass marker: lane 1, cell-free extract (25 µg); lane 2, flow-through (25 µg); lane 3, stringency wash (15 µg); lane 4, elution fraction (2 µg)

F

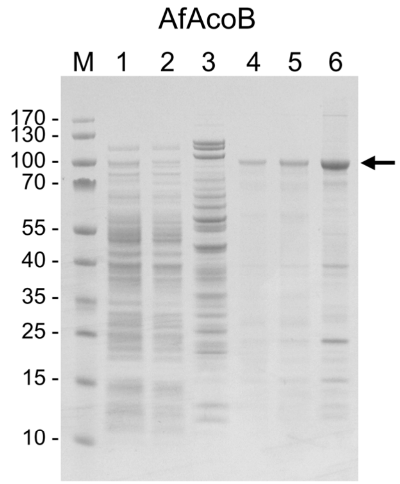

SDS-PAGE analysis of recombinant *A. fumigatus* AcoB purification: M, molecular mass marker: lane 1, cell-free extract (25 µg); lane 2, flow-through (20 µg); lane 3, stringency wash (15 µg); lane 4, elution fraction (2 µg); lane 5, elution fraction (3 µg); lane 6, Resource Q elution (4 µg)

G

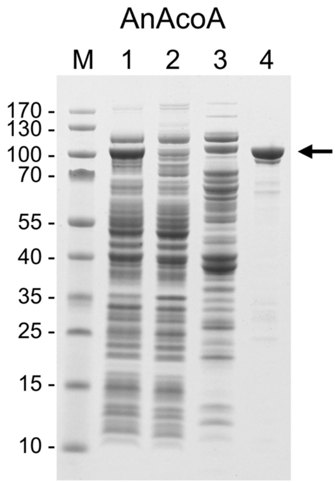

SDS-PAGE analysis of recombinant *A. nidulans* AcoA purification: M, molecular mass marker: lane 1, cell-free extract (25 µg); lane 2, flow-through (20 µg); lane 3, stringency wash (15 µg); lane 4, elution fraction (2 µg)

H

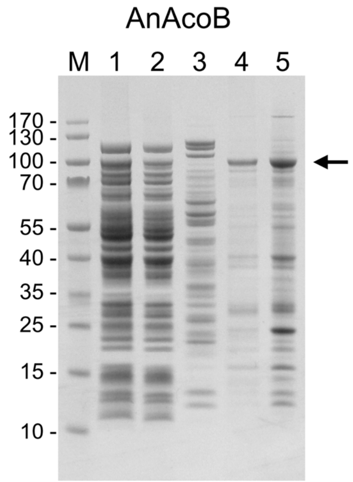

SDS-PAGE analysis of recombinant *A. nidulans* AcoB purification: M, molecular mass marker: lane 1, cell-free extract (25 µg); lane 2, flow-through (20 µg); lane 3, stringency wash (8 µg); lane 4, elution fraction (3 µg); lane 5, Resource Q elution (approx. 8 µg)

I

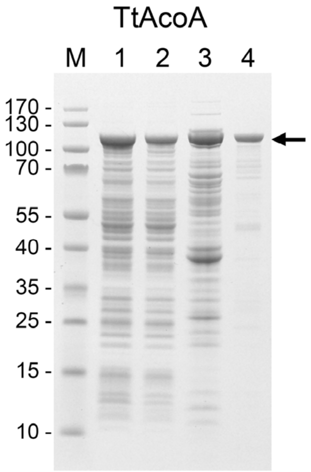

SDS-PAGE analysis of recombinant *T. thermophilus* AcoA purification: M, molecular mass marker: lane 1, cell-free extract (25 µg); lane 2, flow-through (20 µg); lane 3, stringency wash (15 µg); lane 4, elution fraction (2 µg)

J

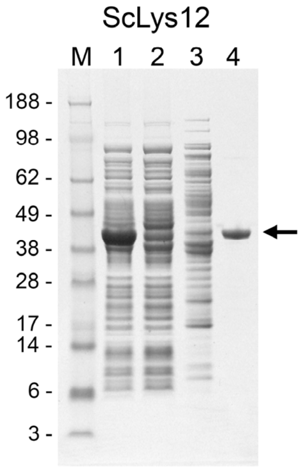

SDS-PAGE analysis of recombinant *S. cerevisiae* Lys12 purification: M, molecular mass marker: lane 1, cell-free extract (25 µg); lane 2, flow-through (20 µg); lane 3, stringency wash (15 µg); lane 4, elution fraction (2 µg)

K

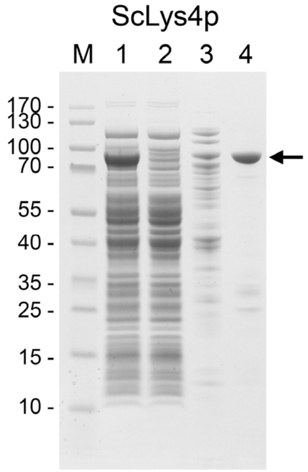

SDS-PAGE analysis of recombinant *S. cerevisiae* Lys4 purification: M, molecular mass marker: lane 1, cell-free extract (25 µg); lane 2, flow-through (25 µg); lane 3, stringency wash (10 µg); lane 4, elution fraction (2 µg);

L

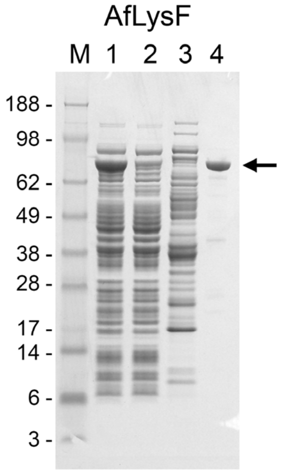

SDS-PAGE analysis of recombinant *A. fumigatus* LysF purification: M, molecular mass marker: lane 1, cell-free extract (25 µg); lane 2, flow-through (25 µg); lane 3, stringency wash (15 µg); lane 4, elution fraction (2 µg);

Figure S2

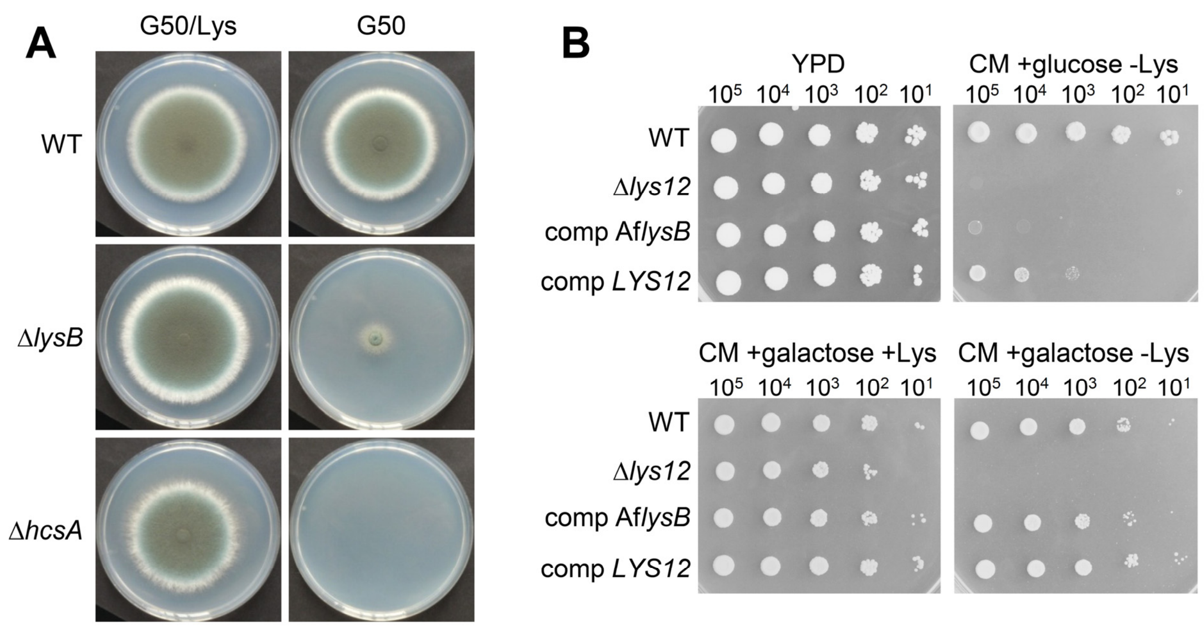

Fig. S2: Growth phenotypes of *A. fumigatus* and *S. cerevisiae* homoisocitrate dehydrogenase mutants in the presence and absence of lysine. A: *A. fumigatus* wild type (WT), homoisocitrate dehydrogenase deletion mutant (Δ*lysB*) and homocitrate synthase deletion mutant (Δ*hcsA*) were grown for four days on glucose containing medium with (G50/Lys) and without (G50) lysine addition. All strains show the same growth morphology in the presence of lysine. In the absence of lysine wild-type growth is unaffected, whereas a homocitrate synthase mutant is unable to grow. Growth of the homoisocitrate dehydrogenase mutants is strongly retarded, but after prolonged incubation a small colony is formed pointing to the existence of another enzyme that can partially take over the function of LysB. **B:** Complementation of a *S. cerevisiae* homoisocitrate dehydrogenase (*lys12*) mutant with the *A. fumigatus lysB* gene. *S. cerevisiae* wild type (WT), *lys12* deletion mutant (Δ*lys12*), a *lys12* mutant carrying the *A. fumigatus lysB* gene under control of the galactose inducible GAL1 promoter on the pYES plasmid (comp Af*lysB*) and a *lys12* mutant complemented with the *LYS12* gene (comp *LYS12*) were sequentially diluted and spotted on various agar plates. All strains grow at similar rates on YPD medium or minimal medium supplemented with lysine. In the absence of lysine the *A. fumigatus lysB* gene complements the *lys12* phenotype when galactose is used for induction of the GAL1 promoter.

Figure S3

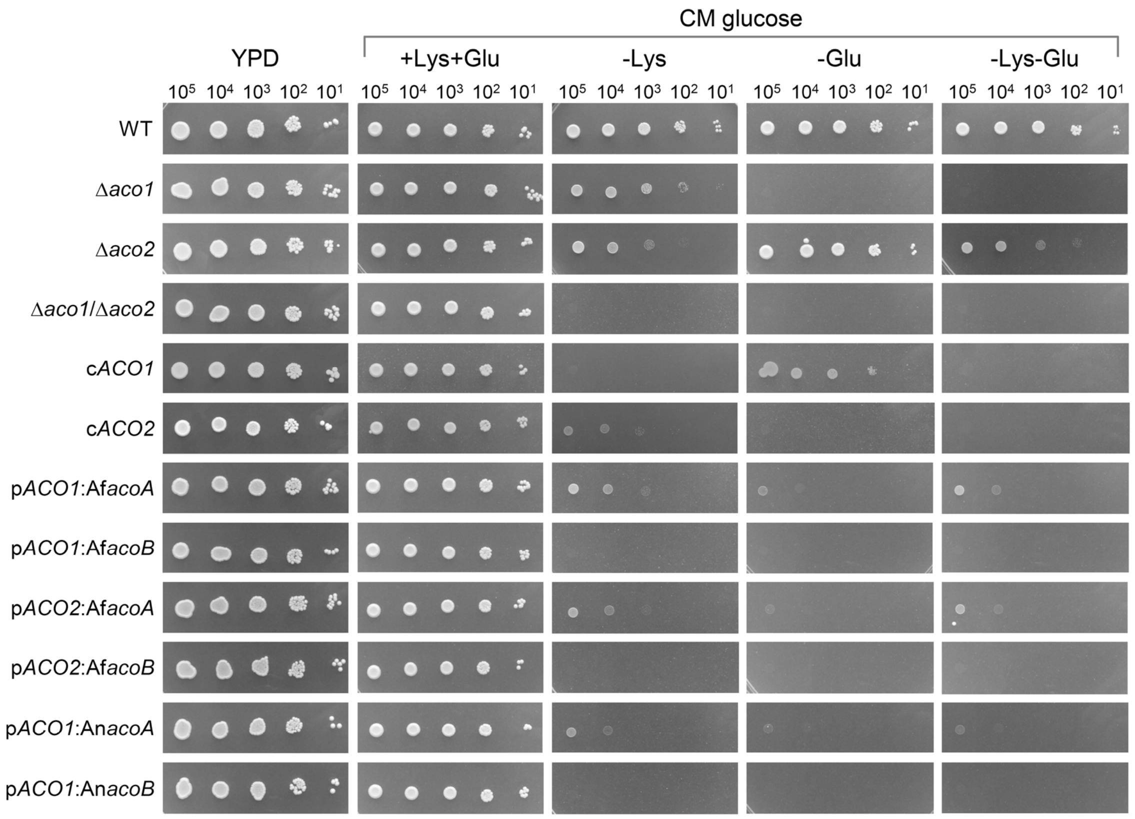

Fig. S3: Growth analysis of *S. cerevisiae* aconitase mutants and complemented strains on solid media in the presence and absence of lysine and glutamate. This figure is supplementary to Fig. 4 from the main manuscript and depicts all *S. cerevisiae* aconitase mutant and complemented strains constructed and tested in this study. WT = wild type; Δ*aco1* = *aco1* deletion mutant; Δ*aco2* = *aco2* deletion mutant; Δ*aco1*/Δ*aco2* = mutant deleted in the *aco1* and *aco2* gene; *cACO1* and *cACO2* = complementation of Δ*aco1*/Δ*aco2* with *S. cerevisiae ACO1* and *ACO2*, respectively; p*ACO1*Af*acoA* and p*ACO1*Af*acoB* = complementation of Δ*aco1*/Δ*aco2* with *A. fumigatus acoA* and *acoB* under the control of the *S. cerevisiae ACO1* promoter; p*ACO2*Af*acoA* and p*ACO2*Af*acoB* = complementation of Δ*aco1*/Δ*aco2* with *A. fumigatus acoA* and *acoB* under the control of the *S. cerevisiae ACO2* promoter; p*ACO1*An*acoA* and p*ACO1*An*acoB* = complementation of Δ*aco1*/Δ*aco2* with *A. nidulans acoA* and *acoB* under the control of the *S. cerevisiae ACO1* promoter. The following media were used: YPD medium; glucose medium supplemented with lysine and glutamate (+Lys +Glu); glucose medium without lysine but with glutamate (-Lys); glucose medium without glutamate but with lysine (-Glu); glucose medium without lysine and without glutamate (-Lys -Glu). Growth phenotypes are identical to those in liquid media shown in Fig. 4. In addition, complementation under control of the *ACO2* promoter is less well pronounced than under control of the *ACO1* promoter, which is in agreement with lower gene expression of *ACO2* as depicted in Fig. 5 in the main manuscript. Additionally, aconitase A, but not aconitase B from *A. nidulans* partially complements amino acid auxotrophies of the Δ*aco1*/Δ*aco2* mutant, although complementation is less well pronounced than with *A. fumigatus acoA*.

Table S1: Half time of inactivation of iron-sulphur cluster containing aconitases and homoaconitases. ScAco1P = *S. cerevisiae* aconitase Aco1p; ScAco2p = *S. cerevisiae* aconitase Aco2p; AfAcoA = *A. fumigatus* aconitase AcoA; AnAcoA = *A. nidulans* aconitase AcoA; TtAcoA = *T. thermophilus* aconitase (aconitate hydratase) AcoA; ScLys4 = *S. cerevisiae* homoaconitase Lys4p; LysF = *A. fumigatus* homoaconitase LysF. All enzymes were purified and reactivated and aliquots shock-frozen in liquid nitrogen. Samples were thawed and incubated on ice. At distinct time points samples were removed and activity was tested in standard assays with either aconitate or homoaconitate as substrate. Each determination was repeated three times. Data show the mean value with standard deviation.

Enzyme	Half time of inactivation [min]
ScAco1p	8.31 ± 0.27
ScAco2p	8.925 ± 2.37
AfAcoA	24.22 ± 0.7
AnAcoA	17.18 ± 2.19
TtAcoA	126.8 ± 31.7
ScLys4p	9.45 ± 1.06
LysF	16.2 ± 1.8

Table S2: ***A. fumigatus*** **and** ***S. cerevisiae*** **strains used in this study.**

Name	relevant genotype	origin
Aspergillus fumigatus		
CBS 144.89	wild type	Centraalbureau voor Schimmelcultures, NL
Δ*akuB*^{Ku80}	Δ*akuB*::*pyrG*	(da Silva Ferreira *et al.*, 2006)
Δ*lysB hph*	Δ*akuB*::*pyrG*; Δ*lysB*::*hph*	this work
Δ*hcsA hph*	Δ*akuB*::*pyrG*; Δ*hcsA*::*hph*	(Schöbel *et al.*, 2010)
Δ*acoB hph*	Δ*akuB*::*pyrG*; Δ*acoB*::*hph*	this work
P*xylP:acoA ptrA*	*ptrA*, P*acoA::*P*xylP*	this work
Saccharomyces cerevisiae		
CLIB 334	wild type	Centre International de Ressources Microbiennes, France
Y00000 (BY4741)	*MAT* a; *his3Δ1; leu2Δ0; met15Δ0; ura3Δ0*	Euroscarf, Frankfurt, Germany (Brachmann *et al.*, 1998)
Y05212 (Δ*aco1*)	BY4741; *MAT* a; *his3Δ1; leu2Δ0; met15Δ0; ura3Δ0*; YLR304c::kanMX4	Euroscarf, Frankfurt, Germany
Y07022 (Δ*aco2*)	BY4741; *MAT* a; *his3Δ1; leu2Δ0; met15Δ0; ura3Δ0*; YJL200c::kanMX4	Euroscarf, Frankfurt, Germany
Y01485 (Δ*lys12*)	BY4741; *MAT* a; *his3Δ1; leu2Δ0; met15Δ0; ura3Δ0*; YIL094c::kanMX4	Euroscarf, Frankfurt, Germany
Y01485 comp *LYS12*	BY4741; *MAT* a; *his3Δ1; leu2Δ0; met15Δ0*; *ura3Δ0*; YIL094c::kanMX4; pYES2 p*GAL1:LYS12*, *URA3*	this work
Y01485 comp Af*lysB*	BY4741; *MAT* a; *his3Δ1; leu2Δ0; met15Δ0*; *ura3Δ0*; YIL094c::kanMX4; pYES2 p*GAL1:*Af*lysB, URA3*	this work
Y05212 Δ*aco2* (Δ*aco1*/Δ*aco2*)	BY4741; *MAT* a; *his3Δ1; leu2Δ0; met15Δ0*; *ura3Δ0*; YLR304c::kanMX4; YJL200c::*URA3*	this work
c*ACO1*	BY4741; *MAT* a; *his3Δ1; leu2Δ0; met15Δ0*; *ura3Δ0*; YJL200c::*URA3*; kanMX4::YIL094c	this work
c*ACO2*	BY4741; *MAT* a; *his3Δ1; leu2Δ0; met15Δ0*; *ura3Δ0*; YLR304c::kanMX4; YJL200c::*URA3*::YJL200c	this work

p*ACO1*:Af*acoA* pYES_HIS3	BY4741; *MAT* a; *his3Δ1; leu2Δ0; met15Δ0*; *ura3Δ0;* YLR304c*::*kanMX4; YJL200c*::URA3*; pYES p*ACO1:*Af*acoA, HIS3*	this work
p*ACO1*:Af*acoB* pYES_HIS3	BY4741; *MAT* a; *his3Δ1; leu2Δ0; met15Δ0*; *ura3Δ0;* YLR304c*::*kanMX4; YJL200c*::URA3*; pYES p*ACO1:*Af*acoB, HIS3*	this work
p*ACO2* Af*acoA* pYES_HIS3	BY4741; *MAT* a; *his3Δ1; leu2Δ0; met15Δ0*; *ura3Δ0;* YLR304c::kanMX4; YJL200c:: *URA3*; pYES p*ACO2:*Af*acoA, HIS3*	this work
p*ACO2* Af*acoB* pYES_HIS3	BY4741; *MAT* a; *his3Δ1*; *leu2Δ0; met15Δ0*; *ura3Δ0;* YLR304c*::*kanMX4; YJL200c*::URA3*; pYES p*ACO2:*Af*acoB, HIS3*	this work
p*ACO1* An*acoA* pYES_HIS3	BY4741; *MAT* a; *his3Δ1; leu2Δ0; met15Δ0*; *ura3Δ0;* YLR304c::kanMX4; YJL200c:: *URA3*; pYES p*ACO1:*An*acoA, HIS3*	this work
p*ACO1* An*acoB* pYES_HIS3	BY4741; *MAT* a; *his3Δ1; leu2Δ0; met15Δ0*; *ura3Δ0;* YLR304c::kanMX4; YJL200c*::URA3*; pYES p*ACO1:*An*acoB, HIS3*	this work

Table S3: Oligonucleotides used in this study. Names of oligonucleotides indicate the target gene and restrictions sites (where applicable). Restriction or mutation sites in sequences are shown in bold letters.

Number	Name	Sequence (5´- 3´)	Description
**Recombinant overproduction of enzymes**			
P1	Aco1_Bam_f	**GGA TCC** AGA GAT TCA AAA GTC AAC C	recombinant overproduction of *S. cerevisiae* Aco1p
P2	Aco1_Not_r	**GCG GCC GC**T TAT TTC TTC TCA TCG GCC	recombinant overproduction of *S. cerevisiae* Aco1p
P3	Aco2_Bgl_f	**AGA TCT** CCC TCT GTA TCT AAA AAT TTT C	recombinant overproduction of *S. cerevisiae* Aco2p
P4	Aco2_Not_r	**GCG GCC GC**T TAT TCG TTT CTT CGT ATA TTA C	recombinant overproduction of *S. cerevisiae* Aco2p
P5	AfAcoA-Bam-f2	**GGA TCC** CTC GAC AAG AAG GTC GAG ATG	recombinant overproduction of *A. fumigatus* AcoA
P6	NotAcoAAf_down	**GCG GCC GC**C TAT TTG GCA CCG GAC TTG C	recombinant overproduction of *A. fumigatus* AcoA
P7	Bam_AfAcoB_f	**GGA TCC** TAC GCC CAT TTG GAC AAT GC	recombinant overproduction of *A. fumigatus* AcoB
P8	Not_AfAcoB_r	**GCG GCC GC**T CAC TGG TGT GCC CGC TTG	recombinant overproduction of *A. fumigatus* AcoB
P9	AcoAAnBam_for	**GGA TCC** ACC GTT GCC GAC CTC G	recombinant overproduction of *A. nidulans* AcoA
P10	AnAcoANot_rev2	**GCG GCC GC**G TTT AGT TGC CAG ACT TGC G	recombinant overproduction of *A. nidulans* AcoA
P11	Bam_AnAcoB_f	**GGA TCC** TAC TCT CAT CTT GAC AAT GC	recombinant overproduction of *A. nidulans* AcoB
P12	Not_AnAcoB_r	**GCG GCC GC**C TAC CTC TTG GAA AGC ACG	recombinant overproduction of *A. nidulans* AcoB
P13	Bgl_TTherAco_f	**AGA TCT** ATG AAG AAC AGC TTC C	recombinant overproduction of *T. thermophilus* AcoA

P14	Not_TTherAco_r	**GCG GCC GC**T TAC TCC GTG GCC TTG	recombinant overproduction of *T. thermophilus* AcoA
P15	Lys4_Bam_f	**GGA TCC** AAA GGT CAG AAC CTA ACT G	recombinant overproduction of *S. cerevisiae* Lys4p
P16	Lys4_Not_r	**GCG GCC GC**T TAT AGT TGG GAT TTG ACC	recombinant overproduction of *S. cerevisiae* Lys4p
P17	Bam_HicDHsc_f	**GGA TCC** ATG TTT AGA TCT GTT GCT ACT AG	recombinant overproduction of *S. cerevisiae* Lys12p
P18	Not_HicDHsc_r	**GCG GCC GC**C TAT AAT CTC GAC AAA AC	recombinant overproduction of *S. cerevisiae* Lys12p
P19	LysF_Bam_w/o1int_f	**GGA TCC** GAG GCA TCA TCC TCC ACG AC	recombinant overproduction of *A. fumigatus* LysF
P20	LysFHind_cDNA_re	**AAG CTT** TCA AGC ATT TCT AAT CTC C	recombinant overproduction of *A. fumigatus* LysF
**Deletion of *A. fumigatus* Homoisocitrate Dehydrogenase *lysB***			
P21	HindHicDHDelUpF	**AAG CTT** GAG CAC TAC TTG TTA GTG TC	*A. fumigatus hcdA* deletion
P22	NotHicDHDelUpR	CAG CCT CCA TCA T**GC GGC CGC** TGT GAG CTT CGG GTG TTG TG	*A. fumigatus hcdA* deletion
P23	HindHicDHDelDoR	**AAG CTT** ACG ATC AGG AAA CAA GGC TAC	*A. fumigatus hcdA* deletion
P24	NotHicDHDelDoF	GAA GCT CAC A**GC GGC CGC** ATG ATG GAG GCT GCG ATG G	*A. fumigatus hcdA* deletion
**Deletion of *A. fumigatus* Aconitase B *acoB***			
P25	AcoA2DelNotUp_r	GTA TCG ATA **GCG GCC GC**T TGA CGG CCT GTG GAA G	*A. fumigatus acoB* deletion
P26	AcoA2DelBamUp_f	**GGA TCC** AGC GTG TAA GGA TTT AAG CAC	*A. fumigatus acoB* deletion
P27	AcoA2DelNotDo_f	CGT CAA **GCG GCC GC**T ATC GAT ACC GGG TGA C	*A. fumigatus acoB* deletion
P28	AcoA2DelBamDo_r	**GGA TCC** GTG CTC AGC CAG ATG GAC	*A. fumigatus acoB* deletion

**Complementation of a *S. cerevisiae lys12* mutant**			
P29	MitoLys12_whole	GGG AAT ATT AAG CTT GGT ACC GAG CTC ATG TTT AGA TCT GTT GCT ACT AGA TTA TCT GCC TGC CGT GGG TTA GCA TCT AAC GCT GCT CGC AAA GGA TCC GCT GCT AGG ACT CTT CGC	complementation with *A. fumigatus hcdA*
P30	MitoLysF_HicDH_r	CTA ATT ACA TGA TGC GGC CCT CTA GAT GCA TGC TCG AGC GGC CGC TTA CAG TCT CTT GAG CAC ATC	complementation with *A. fumigatus hcdA*
P31	ScHicDHBam_for	**GGA TCC** ATG TTT AGA TCT GTT GCT ACT AG	complementation with *S. cerevisiae LYS12*
P32	ScHicDGNot_rev	**GCG GCC GC**C TAT AAT CTC GAC AAA ACG TCG TC	complementation with *S. cerevisiae LYS12*
**Generation of a *S. cerevisiae aco1*/*aco2* double deletion mutant**			
P33	Del-ScAco2_f	GAC TTT CTT TAA ACA TAA AAC TCT AAG AGC AAT AAG CAA GAG ATT CGA TGT CGA AAG CTA CAT ATA AGG	deletion of *ACO2* in Y05212 strain
P34	Del-ScAco2_r	GAC TAT TAA ATC GGA CTC TTA ATT TTT AGC TAA TAT CTC TTC AGA ATT TAG TTT TGC TGG CCG CAT C	deletion of *ACO2* in Y05212 strain
P35	Aco2-probe-f	GAC ATA TCT AAA GGA TTG CAC	southern blot probe *S. cerevisiae ACO2* gene
P36	Aco2-probe-r	CTC TTG CTT ATT GCT CTT AGA G	southern blot probe *S. cerevisiae ACO2* gene
**Generation of the 2 µm plasmid pYES_HIS3**			
P37	His3-pYes-f	GTA TTC TTA ACC CAA CTG CAC AGA ACA AAA ACC TGC AGG AAA CGA AGA TAA ATC ATG ACA GAG CAG AAA GCC	*HIS3*-marker in pYES complementation vector
P38	His3-pYes-pro-r	GCT CTA ATT TGT GAG TTT AGT ATA CAT GCA TTT ACT TAT AAT ACA GTT TTC TAC ATA AGA ACA CCT TTG G	*HIS3*-marker in pYES complementation vector
**Complementation of the *S. cerevisiae aco1*/*aco2* double deletion mutant with *Aspergillus* aconitase genes**			
P39	comp_AcoA_f	**GGA TCC** ATG CTG TCT GCA CGT TCT GCC ATC AAG AGA CCC ATT GTT CGT GGT CTT GCG ACA GTC TCC AAC TTG ACT CTC GAC AAG AAG GTC GAG	amplification of *A. fumigatus acoA*
P40	comp_AcoA_r	**GCG GCC GC**C TAT TTG GCA CCG GAC TTG	amplification of *A. fumigatus acoA*

P41	comp_AcoB_f	**GGA TCC** ATG CTG TCT GCA CGT TCT GCC ATC AAG AGA CCC ATT GTT CGT GGT CTT GCG ACA GTC TCC AAC TTG ACT TAC GCC CAT TTG GAC AAT GC	amplification of *A. fumigatus acoB*
P42	comp_AcoB_r	**GCG GCC GC**T CAC TGG TGT GCC CGC TTG	amplification of *A. fumigatus acoB*
P43	Seq_pYES_f	CTA GCA GCT GTA ATA CGA CTC	sequencing of *acoA*/*acoB* integration in pYES
P44	Seq_pYES_r	CTC TAG ATG CAT GCT CGA G	sequencing of *acoA*/*acoB* integration in pYES
P45	pAco1_r	GTC AAG TTG GAG ACT GTC G	amplification *S. cerevisiae ACO1* promoter
P46	pAco1_f	GTA ACC ACC ACA CCC GCC GCG CTT AAT GGG GCG CTA CAG GGC GCG TGG GGA TGA TCC ACT AGT CTA TGT TAG GAA GCG GTC TC	amplification *S. cerevisiae ACO1* promoter
P47	cAcoA_aco2_f	GCT ACA GGG CGC GTG GGG ATG ATC CAC TAG TCA TAT GTT TCG TTC CTA TTA CAC CAT ATG TTT CGT TCC TAT TAC AC	amplification *S. cerevisiae ACO2* promoter
P48	cAcoA_aco2_r	GTT GAT GTA GTT ACC CTT CTC AGT GTT GGC CAT CTC GAC CTT CTT GTC GAG GAA CAT ATT GGC ATG TGT TG	amplification *S. cerevisiae ACO2* promoter with Af*acoA* site
P49	AfAcoApAco2_f	GTT TTA TAT AAA GAG GCA TTT GGC AAC ACA TGC CAA TAT GTT CCT CGA CAA GAA GGT CGA GAT GGC CAA C	amplification of *A. fumigatus acoA* with p*ACO2* complementing sites
P50	AfAcoApAco2_r	CAT AAC TAA TTA CAT GAT GCG GCC CTC TAG ATG CAT GCT CGA GCG GCC GCC TAT TTG GCA CCG GAC TTG C	amplification of *A. fumigatus acoA* with pYES complementing sites
P51	cAcoB_aco2_r	CAT TGT TAG TGC CCG TCA ACA GGG ACT CCT CCG CAT TGT CCA AAT GGG CGT AGA ACA TAT TGG CAT GTG TTG	amplification *S. cerevisiae Aco2* promoter with Af*acoB* site
P52	AfAcoBpAco2_f	GTT TTA TAT AAA GAG GCA TTT GGC AAC ACA TGC CAA TAT GTT CTA CGC CCA TTT GGA CAA TGC GGA G	amplification of *A. fumigatus acoB* with p*aco2* complementing sites
P53	AfAcoBpAco2_r	CAT AAC TAA TTA CAT GAT GCG GCC CTC TAG ATG CAT GCT CGA GCG GCC GCT CAC TGG TGT GCC CGC TTG G	amplification of *A. fumigatus acoB* with pYES complementing sites
P54	AnAcopAco1_f	CGC GCT TAA TGG GGC GCT ACA GGG CGC GTG GGG ATG ATC CAC TAG TCT ATG TTA GGA AGC GGT CTC	amplification *S. cerevisiae Aco1* promoter
P55	AnAcoApAco1_r	CTT TTC GAG ATT GCA CAT CTC GAC CTT CTT GTC GAG GTC GGC AAC GGT GGC CAT AGT CAA GTT GGA GAC TGT C	amplification *S. cerevisiae Aco1* promoter with An*acoA* site

P56	AnAcoBpAco1_r	CAT TGT TCG TAC CAG TCA AAA GTG ACT CCT CCG CAT TGT CAA GAT GAG AGT AAG TCA AGT TGG AGA CTG TC	amplification *S. cerevisiae Aco1* promoter with An*acoB* site
P57	pAco1_AnAcoA_f	CTG CCA TCA AGA GAC CCA TTG TTC GTG GTC TTG CGA CAG TCT CCA ACT TGA CTA TGG CCA CCG TTG CCG AC	amplification of *A. nidulans acoA* with p*aco1* complementing sites
P58	pAco1_AnAcoA_r	CAT AAC TAA TTA CAT GAT GCG GCC CTC TAG ATG CAT GCT CGA GCG GCC GCT TAG TTG CCA GAC TTG CGG	amplification of *A. nidulans acoA* with pYES complementing sites
P59	pAco1_AnAcoB_f	CCA TCA AGA GAC CCA TTG TTC GTG GTC TTG CGA CAG TCT CCA ACT TGA CTT ACT CTC ATC TTG ACA ATG CG	amplification of *A. nidulans acoB* with p*aco1* complementing sites
P60	pAco1_anacoB_r	CAT AAC TAA TTA CAT GAT GCG GCC CTC TAG ATG CAT GCT CGA GCG GCC GCC TAC CTC TTG GAA AGC ACG	amplification of *A. nidulans acoB* with pYES complementing sites
P61	AnAcoAmi_up	GAG AGA GGT GGT GGC ACC	control *A. nidulans acoA*
P62	AfAcoA_mi_rev	CGT CCT TGT CCT TCA GCT TG	control *A. fumigatus acoA*
P63	pAco2_f	CTA AGA GCA ATA AGC AAG AGA TTC	control *S. cerevisiae ACO2* promoter
P64	AfAcoA2_Not_r	**GCG GCC GC**T CAC TGG TGT GCC CGC TTG	control *A. fumigatus acoB*
**Complementation of *aco1*/*aco2* deletion mutant with *S. cerevisiae ACO1* and *ACO2***			
P65	comp_Aco1_f	GAT ATT GTC CCA GGT GGT ATC	amplification of *ACO1* with promoter fragment
P66	comp_Aco1_r	GGA AAA CTT CGC GGG ATT TG	amplification of *ACO1* with promoter fragment
P67	comp_Aco2_f	GTC GCT ATT GAG TGT TTT GTG	amplification of *ACO2* with promoter fragment
P68	comp_Aco2_r	CCT CCT TCA TCC GTA TCA TC	amplification of *ACO2* with promoter fragment
P69	nes_comp1_f	GAA GCA GTA CCA TTC AAG CTG	amplification of *ACO1* with promoter fragment
P70	nes_comp1_r	GAT CTA AAA AGC CAA ATT ACA AG	amplification of *ACO1* with promoter fragment
P71	nes_comp2_f	GAT AAT TGT TGG GAT TCC GTT G	amplification of *ACO2* with promoter fragment

P72	nes_comp2_r	GTC GTC GCC AAC CAG ATA CAG	amplification of *ACO2* with promoter fragment
P73	Aco1-probe-f	GTC TTC TGT ACA CCG TAC C	southern blot probe *S. cerevisiae* *ACO1* gene
P74	Aco1-probe-r	CTA CAT TAT CAA TCC TTG CAC	southern blot probe *S. cerevisiae* *ACO1* gene
**Site directed mutation of *S. cerevisiae ACO1* and *ACO2***			
P75	ScAco1MutR_r	CTA AAT GAC C**Tt T**GT ATT TCA ACC ATG	amplification *N*-terminal fragment *ACO1*
P76	ScAco1MutR_f	CAT GGT TGA AAT AC**A aA**G GTC ATT TAG	amplification *C*-terminal fragment *ACO1*
P77	ScAco2MutK_r	GAT GGC C**Tc T**AT ATT TCA ACC AG	amplification *N*-terminal fragment *ACO2*
P78	ScAco2MutK_f	CTG GTT GAA ATA T**Ag A**GG CCA TC	amplification *C*-terminal fragment *ACO2*
**qRT-PCR oligonucleotides**			
P79	RTAcoA_f	CAG GGT ATG CTT CCT CTC AC	qRT-PCR *A. fumigatus acoA*
P80	RTAcoA_r	GTC GAA GGG AGC GCT G	qRT-PCR *A. fumigatus acoA*
P81	RTAcoB_f	CTG AAG AAG CAG GGT GTT GTC	qRT-PCR *A. fumigatus acoB*
P82	RTAcoB_r	CTC TTC TTG GTG ACC TGC AG	qRT-PCR *A. fumigatus acoB*
P83	RT_AcoC_f	CCT TCG CCT TCA TCT TTC AAC	qRT-PCR *A. fumigatus acoC*
P84	RT_AcoC_r	CAA ACC TGA GCC CCT CAA TG	qRT-PCR *A. fumigatus acoC*
P85	RT_LysF_f	CAG CCG CAA TGC CGT CAA C	qRT-PCR *A. fumigatus lysF*
P86	RT_LysF_r	GAC AGT GAC CTG GGA AGT G	qRT-PCR *A. fumigatus lysF*

P87	RT_Tub_f	GAT TCC CAA CAA CAT CCA GAC	qRT-PCR *A. fumigatus β-tub*
P88	RT_Tub_r	GAA AGC CTT GCG ACG GAA C	qRT-PCR *A. fumigatus β-tub*
P89	RT_Aco1_f	CAA AGG TGT TCC AGA CAC TG	qRT-PCR *S. cerevisiae ACO1*
P90	RT_Aco1_r	GAG CGA AAG ACT TTG TGA TG	qRT-PCR *S. cerevisiae ACO1*
P91	RT2_ScAco2_f	CTT ATG ACC TTG ACG GAA CTG	qRT-PCR *S. cerevisiae ACO2*
P92	RT2_ScAco2_r	CTT GGT GAC AAA GCA GCA TG	qRT-PCR *S. cerevisiae ACO2*
P93	RT_ScTub2_f	CGT TGC AGC CTT CTT TAG AG	qRT-PCR *S. cerevisiae TUB2*
P94	RT_ScTub2_r	CAT GTC CAA ACC TTG AGG AG	qRT-PCR *S. cerevisiae TUB2*
**Replacement of the *A. fumigatus acoA* promoter by the *P. chrysogenum xylP* promoter**			
P95	AfAcoAupHind_f	**AAG CTT** GGT ATG GGC GAC CAG G	amplification *A. fumigatus acoA* 5' upstream region
P96	AfAcoAupHigu_r	CCA G**GG CGG CCG C**AA CTG GAC AAT GCT AGG AC	amplification *A. fumigatus acoA* 5' upstream region
P97	XylP_Higu_r	CTT GTG GAG ATC ATG TTG GTT CTT CGA GTC G	amplification *P. chrysogenum xylP* promoter
P98	XylP_HigNot_f	CCA GAA **GCG GCC GC**C TGG TCA TTA TAC CGT TG	amplification *P. chrysogenum xylP* promoter
P99	HiguAcoAAf_f	GAA CCA ACA TGA TCT CCA CAA GGC TTG C	amplification *A. fumigatus acoA* fragment
P100	AfAcoA_Hind_r	GT**A AGC TT**G ACA CCA ATC ACC	amplification *A. fumigatus acoA* fragment
P101	ContXylP_f	CAG CTG GAA CTG TGT AAT AGG	internal control primer in the *P. chrysogenum xylP* promoter
P102	AfAcoA_mi_r	CAC CCT TGA CAG TGA GAA G	control primer within the *A. fumigatus acoA* coding region

Table S4: Accession numbers of protein sequences used for phylogenetic analysis.

Species	Protein	Accession number
**Aconitase**		
Aspergillus clavatus NRRL 1	AcoA	XP_001268345.1
Aspergillus clavatus NRRL 1	AcoB	XP_001269477.1
Aspergillus fumigatus Af293	AcoA	XP_751171.1
Aspergillus fumigatus Af293	AcoB	XP_750430.1
Aspergillus nidulans FGSC A4	AcoA	XP_663129.1
Aspergillus nidulans FGSC A4	AcoB	XP_661498.1
Azotobacter vinelandii DJ	Aco	YP_002799186.1
Bacillus subtilis subsp. *subtilis* str. 168	Aco	NP_389683
Bos taurus	Aco	NP_776402.1
Candida albicans SC5314	Aco1	CAWG_02112.1
Candida albicans SC5314	Aco2	CAWG_01900.1
Coprinopsis cinerea okayama 7#130	Aco1	XP_001834931.2
Coprinopsis cinerea okayama 7#130	Aco2	XP_002912138.1
Cryptococcus neoformans var. neoformans JEC21	Aco1	XP_570245.1
Cryptococcus neoformans var. neoformans JEC21	Aco2	XP_568476.1
Escherichia coli 536	AcnA	YP_669241.1
Fusarium graminearum (*Gibberella zeae* PH-1)	AcoA	XP_388129.1
Fusarium graminearum (*Gibberella zeae* PH-1)	AcoB	XP_390374.1
Homo sapiens	Aco	AAB38416.1
Kluyveromyces lactis NRRL Y-1140	Aco1	XP_452974.1
Kluyveromyces lactis NRRL Y-1140	Aco2	XP_452349.1
Mycobacterium tuberculosis H37Rv	Aco	NP_215991.1
Neurospora crassa OR74A	AcoA	XP_959787.1
Neurospora crassa OR74A	AcoB	XP_961070.1
Pseudomonas aeruginosa 2192	Aco	YP_002441350.1
Pyrococcus furiosus DSM 3638	Aco	NP_577930.1
Saccharomyces cerevisiae	Aco1	NP_013407.1
Saccharomyces cerevisiae	Aco2	NP_012335.1
Scheffersomyces stipitis CBS 6054	Aco1	XP_001386080
Scheffersomyces stipitis CBS 6054	Aco2	XP_001385589.2
Staphylococcus aureus subsp. aureus Mu3	Aco	YP_001441928.1
Sus scrofa Aco2	Aco	NP_999119.1
Thermus thermophilus HB27	Aco	YP_004349.1
Ustilago maydis 521	Aco1	XP_759046.1
Ustilago maydis 521	Aco2	XP_758838.1
**Homoaconitase**		
Aspergillus clavatus NRRL 1	Lys	XP_001274165.1
Candida albicans SC5314	Lys	XP_717250.1
Coprinopsis cinerea okayama 7#130	Lys	XP_002911394.1
Cryptococcus neoformans var. neoformans JEC21	Lys	XP_567622.1
Fusarium graminearum (*Gibberella zeae* PH-1)	Lys	XP_391125.1
Neurospora crassa OR74A	Lys	XP_958836.1
Pyrococcus horikoshii OT3	LysL+S	NP_143565.1+NP_143564.1
Scheffersomyces stipitis CBS 6054	Lys	XP_001383888.2

Thermus thermophilus HB27	LysL+S	BAA74762+YP_005515.1
Ustilago maydis 521	Lys	XP_760939.1
Saccharomyces cerevisiae	Lys4	NP_010520.1
Aspergillus fumigatus Af293	LysF	XP_753748.1
Aspergillus nidulans FGSC A4	LysF	XP_664125.1
Kluyveromyces lactis NRRL Y-140	LysF	XP_452878.1
Methanocaldococcus jannaschii	LysL+S	NP_247997.1+NP_248267.1
**3-Isopropylmalate dehydratase**		
Aspergillus fumigatus Af293	Iso	XP_755459.1
Aspergillus nidulans FGSC A4	Iso	XP_663490.1
Cryptococcus neoformans var. neoformans JEC21	Iso	XP_566619.1
Fusarium graminearum (*Gibberella zeae* PH-1)	Iso	XP_389765.1
Neurospora crassa OR74A	Iso	XP_957462.1
Saccharomyces cerevisiae (strain: S288C)	Iso	NP_011506.1
Ustilago maydis 521	Iso	XP_762157.1

Detailed description of cloning procedures and strain constructions

Generation of **A. fumigatus** *homoisocitrate dehydrogenase and aconitase B deletion mutants*

For deletion of the homoisocitrate dehydrogenase coding region, flanking upstream and downstream regions were amplified from genomic DNA of *A. fumigatus* CBS144.89 using oligonucleotides P21 + P22 and P23 + P24, respectively. Similarly, for deletion of aconitase B oligonucleotides P25 + P26 and P27 + P28 were used for amplification of upstream and downstream flanking regions. Oligonucleotides P22, P24 and P25, P27, respectively, contained overlapping sequences including a *Not*I restriction site that allowed annealing of respective related fragments and elongation by BioTaq red polymerase (Bioline GmbH, Luckenwalde, Germany). After four cycles the flanking oligonucleotides (P21 and P23 for *lysB*, P26 and P28 for *acoB*) were added and amplification was continued by additional 30 PCR cycles. The resulting PCR products were cloned into the pCRII-vector (Invitrogen GmbH, Karlsruhe, Germany), excised with *Hind*III (*lysB*) or *Bam*HI (*acoB*) and cloned into a previously *Hind*III or *Bam*HI restricted and dephosphorylated pUC18 vector (MBI Fermentas, St. Leon-Rot, Germany). The resulting plasmids UpDohcdA_puc and UpDoacoB_puc were linearised by *Not*I restriction and the hygromycin B resistance cassette *hph* (Fleck & Brock, 2010) was introduced resulting in plasmids Δ*hcdA*/*hph* and Δ*acoB*/*hph*. The deletion cassette was excised by *Hind*III or *Bam*HI restriction, gel purified, and used for transformation of the *A. fumigatus* strain *ΔakuB*^{KU80}. Transformation of protoplasts was performed as previously described (Weidner *et al.*, 1998) and transformants were selected on AMM-glucose medium supplemented with 0.6 M KCl, 5 mM lysine and 240 µg ml^{-1} hygromycin B. Transformants were checked for lysine auxotrophy and gene deletion was confirmed by Southern analysis (Southern, 1975). For the *lysB* deletion *EcoR*V restricted genomic DNA was blotted on a nylon membrane and hybridised with a digoxigenin-labelled probe directed against the upstream region of the *lysB* gene. Deletion of *acoB* was verified from *Sal*I restricted genomic DNA and detection using a probe directed against the *acoB* downstream region. The probes were generated by PCR with oligonucleotides P21 + P22 and P27 + P28. Bands were visualised using the chemiluminescence substrate CDP-Star as recommended by the manufacturer (Roche Diagnostics GmbH, Mannheim, Germany).

Complementation of **S. cerevisiae lys12** *mutant with the* **A. fumigatus lysB** *gene*

To investigate the function of *A. fumigatus* homoisocitrate dehydrogenase *lysB* gene, a complementation approach of a *S. cerevisiae lys12* mutant was used. The *lysB* gene was amplified with Phusion polymerase from *A. fumigatus* cDNA using sequence specific oligonucleotides P29 and P30. Since the *lysB* gene from *A. fumigatus* lacked a mitochondrial import sequence, oligonucleotide P29 contained an in frame sequence coding for the

mitochondrial import sequence from *S. cerevisiae* Lys12p. The resulting PCR fragment was cloned into pJET1.2, excised with *Sac*I and *Not*I and cloned into a previously restricted pYES2 vector (Invitrogen GmbH) for gene expression under control of the *GAL1* promoter. As a complementation control, the *LYS12* gene was amplified from genomic DNA of *S. cerevisiae* strain CLIB334 using oligonucleotides P31 and P32. The fragment was subcloned into pJET1.2, excised with *Bam*HI and *Not*I and subcloned into the pYES2 vector. Constructs were transferred to the *S. cerevisiae lys12* mutant Y01485 by heat shock transformation (Yeast Protocols Handbook (March 2001) and transformants were selected on the basis of uracil prototrophy. Several independent transformants were checked for complementation of the lysine auxotrophic phenotype on galactose containing agar plates.

Generation of a* S. cerevisiae aco1/aco2 *double deletion mutant

To generate an *aco1*/*aco2* double deletion mutant, the *aco1* mutant Y05212 was selected as parental strain. As auxotrophic marker for transformation the *URA3* gene was used. The *URA3* gene was amplified from pYES2 with oligonucleotides P33 and P34 that contained flanking regions of the *ACO2* upstream and downstream locus. The PCR fragment was used for heat-shock transformation of strain Y05212 and transformants were selected on the basis of uracil prototrophy. Genomic DNA was isolated from independent transformants and checked by Southern blot analysis. The digoxygenin labelled probe directed against the 5' upstream region of *ACO2* was amplified with GoTaq polymerase (Promega GmbH, Mannheim, Germany) using oligonucleotides P35 and P36. Southern blots were hybridised and developed as recommended by the manufacturer of the chemiluminescent dye CDPstar (Roche Diagnostics GmbH).

***In locus replacement of the* A. fumigatus acoA *promoter by the* xylP *promoter from* P. chrysogenum**

To exchange the native *A. fumigatus acoA* promoter with the xylose inducible *xylP* promoter from *P. chrysogenum* the following PCR fragments were generated: (i) A 642 bp fragment of the *acoA* 5' upstream region (position 1497 – 855 relative to the ATG start codon) was amplified from genomic DNA of *A. fumigatus* strain CBS144.89 with oligonucleotides P95 and P96. The resulting fragment contained a 5' *Hind*III restriction site and a 3' *Not*I restriction site followed by an overlap to the 5' region of the *P. chrysogenum xylP* promoter. (ii) A 1162 bp *xylP* promoter fragment (including the ATG start codon) was amplified from genomic DNA of *P. chrysogenum* strain 844 (DSMZ Braunschweig, Germany) with oligonucleotides P97 and P98. The resulting fragment contained a 5' *Not*I restriction site followed by an overlap to the *acoA* upstream fragment. At its 3' end the fragment was complementary to the 5' coding region of the *acoA* gene. (iii) The first 1161 bp of the *acoA* coding region were amplified with oligonucleotides P99 and P100 from *A. fumigatus* genomic DNA. The 5' oligonucleotide contained an overlap to the 3' end of the *xylP* promoter. The 5' oligonucleotide included a natural *Hind*III restriction site of the *acoA* gene. The *xylP*

promoter was fused by PCR with the *acoA* coding region using oligonucleotides P98 and P100 resulting in fragment p*xylP*:*acoA*$_{code}$. Subsequently, this fragment was fused by PCR with the *acoA* upstream region using oligonucleotides P95 and P100 resulting in *acoA*$_{up}$:p*xylP*:*acoA*$_{code}$. This PCR fragment was cloned into the pJET1.2 cloning vector, excised by *Hind*III restriction and subcloned into the *Hind*III restricted pUC19 vector. The *Not*I restriction site introduced between the fusion of *acoA*$_{up}$ and p*xylP* was used to linearise the plasmid and the *Not*I restricted *ptrA* cassette from plasmid ptrA(Not)-pJet (Fleck & Brock, 2010) was introduced resulting in fragment *acoA*$_{up}$:*ptrA*:p*xylP*:*acoA*$_{code}$. This fragment was released by *Hind*III restriction from the pUC19 vector, gel purified and used for transformation of the *A. fumigatus* Δ*akuB* strain. Transformants were regenerated on osmotically stabilised xylose containing minimal medium with 0.1 µg/ml pyrithiamine. After several rounds of purification genomic DNA was isolated and used for PCR and Southern blot analysis. PCR analyses were performed with oligonucleotides P99 and P102 to confirm an intact *acoA* gene and oligonucleotides P101 and P102 to confirm the *xylP* promoter exchange at the *acoA* locus. For Southern analyses a digoxygenin-labelled probe was generated with oligonucleotides P99 and P100 amplifying the 5' coding region of the *acoA* gene. For fragment size analyses *Xho*I and *EcoR*I digests were made from genomic DNA of the parental strain and transformants.

Generation of the 2 µm plasmid pYES_HIS3

To complement the *S. cerevisiae aco1*/*aco2* double deletion mutant with aconitases from Aspergilli, a 2 µm plasmid with the *HIS3* gene as auxotrophic marker was required. Therefore, a replacement of *URA3* from pYES2 with *S. cerevisiae HIS3* was performed. *HIS3* was amplified from genomic DNA of strain CLIB334 using oligonucleotides P37 and P38 that contained flanking regions of the *URA3* gene in the pYES2 vector. A transformation of strain Y00000 was performed by using a mixture of the pYES2 vector with the PCR product containing the entire *HIS3* gene. Transformants were selected by histidine prototrophy. Plasmids were reisolated from transformants, amplified in *E. coli* DH5α and checked by restriction analysis and re-transformation of various histidine auxotrophic yeast strains. The resulting vector was named pYES_HIS3.

Complementation of the* S. cerevisiae aco1/aco2 *double deletion mutant with* Aspergillus *aconitase genes

For complementation of the *aco1*/*aco2* double deletion mutant with *A. fumigatus* aconitases the aconitase *acoA* was amplified from cDNA and *acoB* from gDNA using oligonucleotides P39 + P40 and P41 + P42, respectively. During amplification the original mitochondrial import sequences of *acoA* and *acoB* was replaced by the mitochondrial import sequence from *S. cerevisiae ACO1* that was encoded on oligonucleotides P39 and P41. PCR products were cloned into pJET1.2, excised with *Bam*HI and *Not*I and subcloned in the previously restricted

pYES_HIS3 vector. Subsequently, the *GAL1* promoter in the resulting plasmids comp_AcoA_pYES_HIS3 and comp_AcoB_pYES_HIS3 was replaced with the *S. cerevisiae ACO1* promoter. Approximately 800 bp of the *ACO1* promoter region were amplified with oligonucleotides P45 and P46 that contained flanking regions of the pYES2 vector and the mitochondrial import sequence of *ACO1*, respectively. The PCR product was mixed with the respective *Bam*HI restricted plasmids comp_AcoA_pYES_HIS3 or comp_AcoB_pYES_HIS3 and used for transformation of the *S. cerevisiae aco1*/*aco2* double deletion mutant. Plasmids were reisolated from resulting strains p*ACO1*:Af*acoA* pYES_HIS3 and p*ACO1*:Af*acoB* pYES_HIS3, amplified in *E. coli* and checked by restriction analyses. For expressing both *A. fumigatus* aconitases under control of the *ACO2* promoter and the *A. nidulans* aconitases under control of the *ACO1* promoter the strategy for plasmid construction was simplified. PCR products were generated that contained the respective promoter with overlap to the region upstream of the *GAL1* promoter in pYES_HIS3 and overlap to the desired aconitase. Additionally, the aconitase of interest was PCR amplified with oligonucleotides overlapping to the desired promoter and to the multiple cloning site of pYES_HIS3. For each plasmid construction the two corresponding PCR products were mixed with *Bam*HI and *Eco*RI restricted pYES_HIS3 and used for transformation of the *aco1*/*aco2* double deletion mutant. The following pairs of PCR products were generated: construction of p*ACO2*:Af*acoA* pYES_HIS3 = P47 + P48 and P49 + P50; construction of p*ACO2*:Af*acoB* pYES_HIS3 = P47 + P51 and P52 + P53; construction of p*ACO1*:An*acoA* pYES_HIS3 = P54 + P55 and P57 + P58; construction of p*ACO1*:An*acoB* pYES_HIS3 = P54 + P56 and P59 + P60. Promoter-gene fusions were confirmed by PCR with oligonucleotides P62 and P63 for p*ACO2*:Af*acoA* pYES_HIS3, P62 and P64 for p*ACO2*:Af*acoB* pYES_HIS3, P46 and P61 for p*ACO1*:An*acoA* pYES_HIS3 and P46 and P60 for p*ACO1*:An*acoB* pYES_HIS3.

***Complementation of* aco1/aco2 *deletion mutant with* S. cerevisiae ACO1 *and* ACO2**

For complementation of the double deletion mutant with *S. cerevisiae ACO1* and *ACO2* in the original genomic locus, both genes were amplified using the sequence specific oligonucleotides P65 + P66 and P67 + P68, respectively. The respective PCR fragments were used as templates for nested PCR with the oligonucleotides P69 + P70 and P71 + P72, respectively. The fragments with specific overlapping regions were directly used for transformation of the *aco1*/*aco2* deletion mutant. Transformants were selected on the basis of lysine prototrophy. Complementation was confirmed by PCR and Southern Blot analysis. Genomic DNA was restricted with *Bgl*II (*ACO1*) or *Eco*RI (*ACO2*) and fragments were detected by sequence specific probes. The *ACO2* upstream probe was generated as already described. A probe directed against the *ACO1* downstream region was amplified by using oligonucleotides P73 + P74.

Supplementary references

Brachmann, C. B., A. Davies, G. J. Cost, E. Caputo, J. Li, P. Hieter & J. D. Boeke, (1998) Designer deletion strains derived from *Saccharomyces cerevisiae* S288C: a useful set of strains and plasmids for PCR-mediated gene disruption and other applications. *Yeast* **14**: 115-132.

da Silva Ferreira, M. E., M. R. Kress, M. Savoldi, M. H. Goldman, A. Härtl, T. Heinekamp, A. A. Brakhage & G. H. Goldman, (2006) The *akuB*$^{(KU80)}$ mutant deficient for nonhomologous end joining is a powerful tool for analyzing pathogenicity in *Aspergillus fumigatus*. *Eukaryot Cell* **5**: 207-211.

Fleck, C. B. & M. Brock, (2010) *Aspergillus fumigatus* catalytic glucokinase and hexokinase: expression analysis and importance for germination, growth, and conidiation. *Eukaryot Cell* **9**: 1120-1135.

Schöbel, F., I. D. Jacobsen & M. Brock, (2010) Evaluation of lysine biosynthesis as an antifungal drug target: biochemical characterization of *Aspergillus fumigatus* homocitrate synthase and virulence studies. *Eukaryot Cell* **9**: 878-893.

Southern, E. M., (1975) Detection of specific sequences among DNA fragments separated by gel electrophoresis. *J Mol Biol* **98**: 503-517.

Weidner, G., C. d'Enfert, A. Koch, P. C. Mol & A. A. Brakhage, (1998) Development of a homologous transformation system for the human pathogenic fungus *Aspergillus fumigatus* based on the *pyrG* gene encoding orotidine 5'-monophosphate decarboxylase. *Curr Genet* **33**: 378-385.
