# Supplementary figures and images for: The fungal α-aminoadipate pathway for lysine biosynthesis requires two enzymes of the aconitase family for the isomerization of homocitrate to homoisocitrate
Source: Mol Microbiol. 2012 Nov 6;86(6):1508–30. doi: 10.1111/mmi.12076 (PMC3556520; doi:10.1111/mmi.12076)

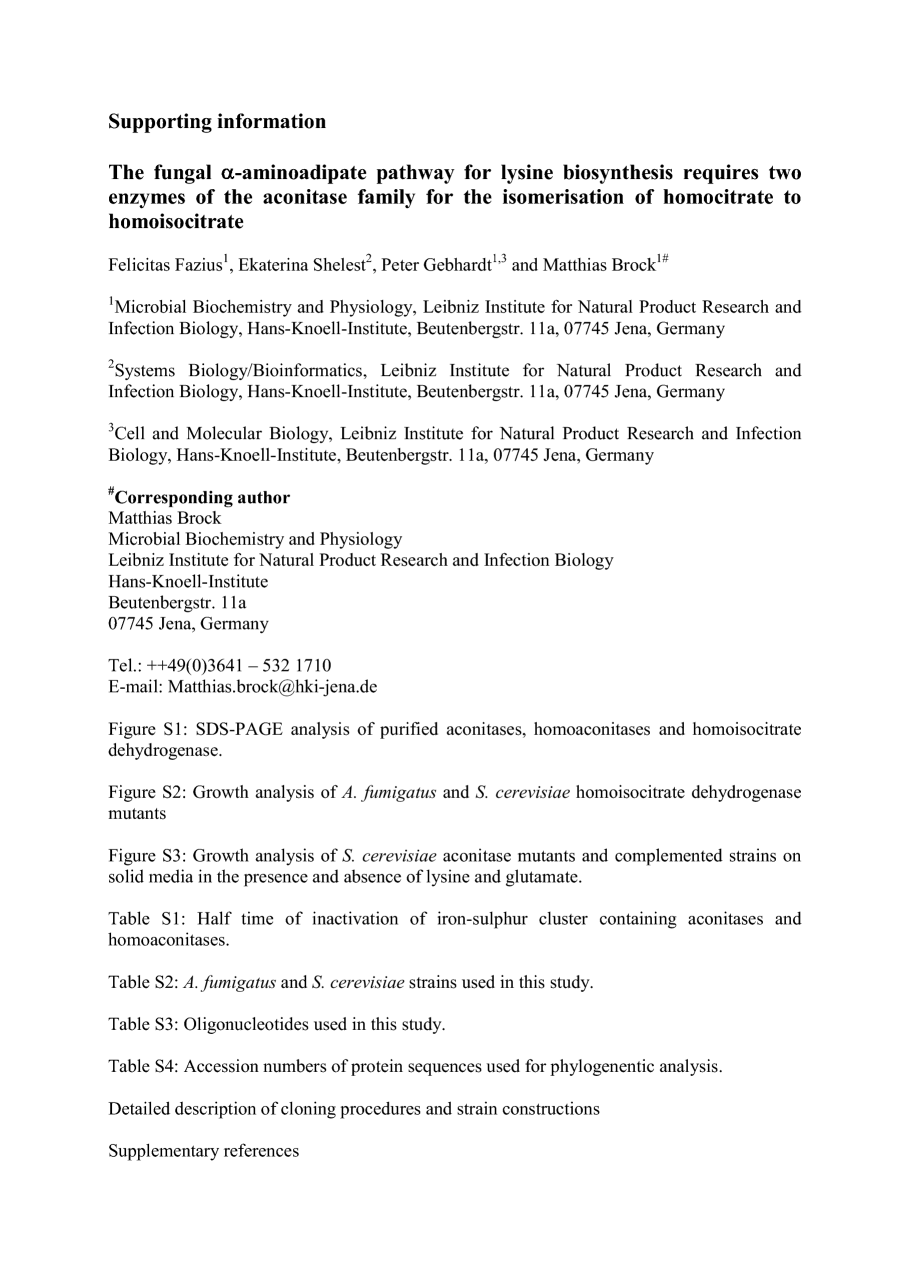

Supplement: Supplementary file 2 [file mmi0086-1508-SD2.png]
